# Are there non-linear relationships between alcohol consumption and long-term health?: a systematic review of observational studies employing approaches to improve causal inference

**DOI:** 10.1186/s12874-021-01486-5

**Published:** 2022-01-14

**Authors:** Rachel Visontay, Matthew Sunderland, Tim Slade, Jack Wilson, Louise Mewton

**Affiliations:** 1 The Matilda Centre for Research in Mental Health and Substance Use, The University of Sydney, Level 6, Jane Foss Russell Building, G02, Sydney, NSW 2006 Australia; 2 Centre for Healthy Brain Ageing, University of New South Wales, Level 1, AGSM (G27), Gate 11, Botany Street, Sydney, NSW 2052 Australia

**Keywords:** Systematic review, Alcohol drinking, Alcohol abstinence, Causality, Risk factors, Protective factors

## Abstract

**Background:**

Research has long found ‘J-shaped’ relationships between alcohol consumption and certain health outcomes, indicating a protective effect of moderate consumption. However, methodological limitations in most studies hinder causal inference. This review aimed to identify all observational studies employing improved approaches to mitigate confounding in characterizing alcohol–long-term health relationships, and to qualitatively synthesize their findings.

**Methods:**

Eligible studies met the above description, were longitudinal (with pre-defined exceptions), discretized alcohol consumption, and were conducted with human populations. MEDLINE, PsycINFO, Embase and SCOPUS were searched in May 2020, yielding 16 published manuscripts reporting on cancer, diabetes, dementia, mental health, cardiovascular health, mortality, HIV seroconversion, and musculoskeletal health. Risk of bias of cohort studies was evaluated using the Newcastle-Ottawa Scale, and a recently developed tool was used for Mendelian Randomization studies.

**Results:**

A variety of functional forms were found, including reverse J/J-shaped relationships for prostate cancer and related mortality, dementia risk, mental health, and certain lipids. However, most outcomes were only evaluated by a single study, and few studies provided information on the role of alcohol consumption pattern.

**Conclusions:**

More research employing enhanced causal inference methods is urgently required to accurately characterize alcohol–long-term health relationships. Those studies that have been conducted find a variety of linear and non-linear functional forms, with results tending to be discrepant even within specific health outcomes.

**Trial registration:**

PROSPERO registration number CRD42020185861.

**Supplementary Information:**

The online version contains supplementary material available at 10.1186/s12874-021-01486-5.

## Background

**Fig. 1:**
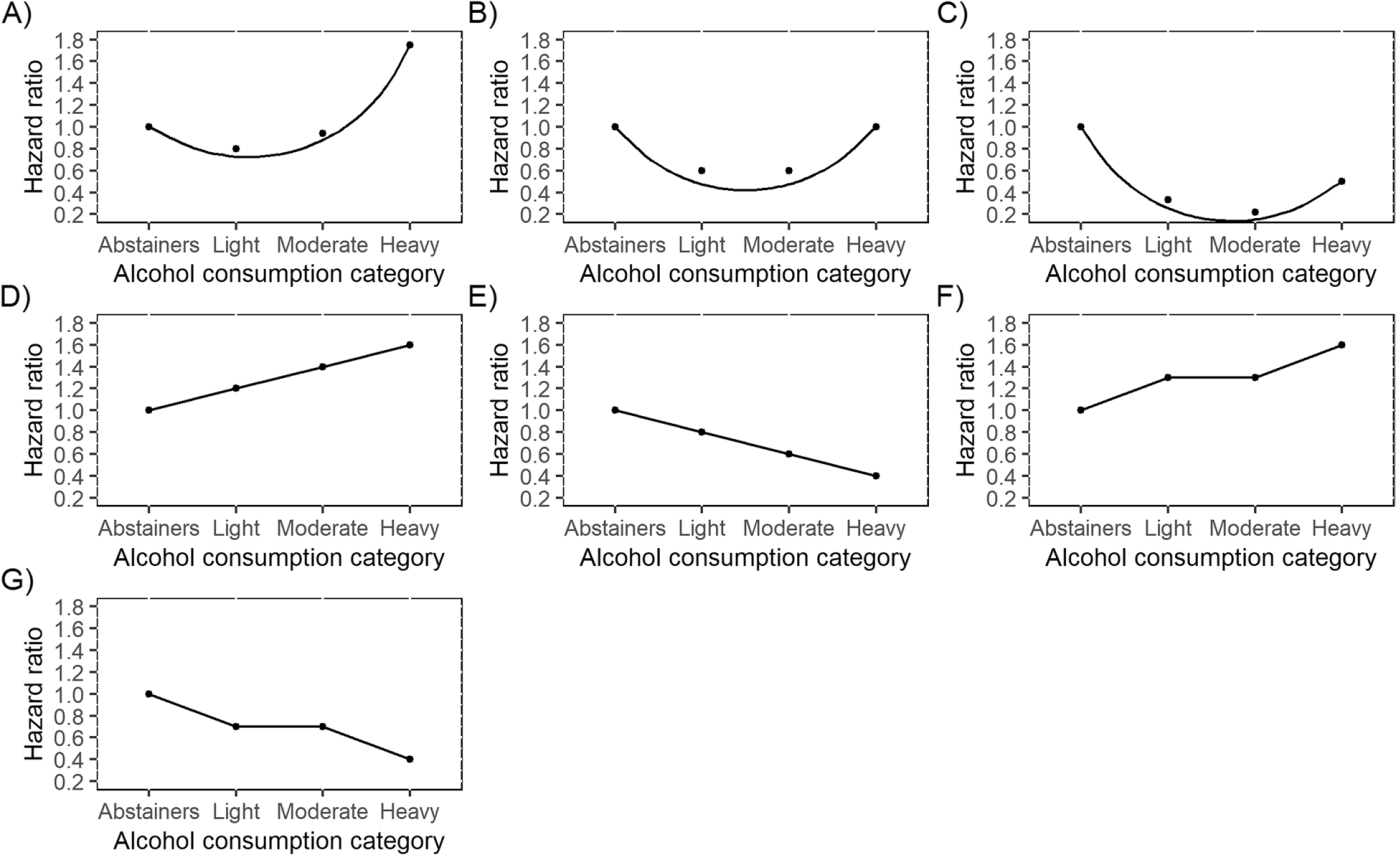
Exemplar functional forms between alcohol consumption and health outcomes. Legend: A = J-shaped; B=U-shaped; C = reverse J-shaped; D = positive linear; E = negative linear; F = monotonically increasing; G = monotonically decreasing

While the contribution of heavy alcohol consumption to the burden of disease is well-known [1], findings that moderate alcohol consumption is associated with health benefits for a wide range of outcomes persist. This relationship is often characterized as ‘J-shaped’, where low-to-moderate consumption coincides with the lowest risk, compared to a slightly higher risk for alcohol abstainers and much greater risk for heavy consumers. However, findings are inconsistent, and many functional forms have been reported for alcohol–long-term health relationships (see Fig. 1 for exemplar forms). Much recent research supports positive linear/monotonically increasing relationships for many outcomes, including most cancers [1, 2]. However, methodologically rigorous individual studies continue to find exceptions, e.g., for mortality [3], and indeed for dementia, diabetes, and particular cardiovascular conditions, evidence remains largely consistent with a J/U-shape [1, 2]. Which of the myriad reported functional forms reflect true causal relationships, and which are merely methodological artefacts, remains unclear.

In addition to these inconsistent findings, J-shaped relationships are sometimes found for certain outcomes (e.g., cirrhosis of the liver) which lack plausible biological mechanisms [4]. More generally, that studies across a broad array of health outcomes (with different underlying biological pathways) arrive at similar functional forms has prompted scrutiny of biases in observational studies – specifically confounding, reverse causality, selection bias and measurement error [5–7]. Confounding may constitute the biggest threat to causal inference, i.e., confidence that a relationship’s observed functional form/strength reflects the actual causal effect of exposure on outcome. Indeed, several confounders (e.g., socioeconomic disadvantage [8, 9] and limited health care access [9]) may be driving the relationship between alcohol abstention and poor health outcomes.

Standard approaches to counter confounding include control for covariates via multivariable regression adjustment, stratification or (exact) matching [10]. These methods are limited; regression with adjustment requires correct specification of the functional form (i.e., algebraic form in regression equations) of covariate–outcome relationships [11, 12], and relies on extreme extrapolation when there are insufficient observations for all combinations of exposure, covariates and outcomes [12–14]. Similarly, the ‘curse of dimensionality’ – that the number of groups to compare increases exponentially with the inclusion of additional covariates, resulting in too few observations per group – is a limitation for matching and stratification [14]. It is also difficult to know if all (and only) relevant covariates have been identified, and whether they may be imperfectly measured, leading to residual confounding. Indeed, some propose that certain relevant confounders are not even measurable [10]. While confounding is generally obviated by the randomization mechanism in randomized controlled trials (RCTs), no long-term RCTs have been performed evaluating the relationships between alcohol use and long-term health outcomes because of ethical and compliance concerns.

To investigate alcohol–long-term health relationships, the field is therefore limited to observational studies. As such, efforts to improve causal inference have centered on mitigating bias. There is increased acknowledgement that data collection and analysis decisions can substantially affect conclusions about relationship strength and form [15, 16], so should be made in a considered, literature-informed manner. A particularly hazardous decision here is treating lifetime abstainers and former drinkers (whose abstinence is often precipitated by illness) as a homogenous group, thereby inducing a ‘sick quitter bias’ which effectively shifts poor health outcomes that have accrued to former drinkers to the abstaining group [7]. Certain tools and strategies can assist with limiting bias, such as creating directed acyclic graphs (DAGs) at study outset, and, following primary analysis, assessing robustness to methodological decisions (‘sensitivity analysis’), bias (‘bias analysis’), sample-specific confounding (‘cross-cohort comparison’) or research type (‘triangulation’) [17]. Indeed, the impact of analytical decisions such as how exposures are categorized and compared has been the focus of recent reviews/meta-analyses of alcohol–health research [15, 16].

Particularly promising, however, for addressing the identified limitations of existing research, are modern methods for data analysis and alternative observational designs. Conventional designs (e.g., prospective cohort studies) can be enhanced with modern analysis methods, such as propensity scores used for matching or weighting, and ‘G-methods’ such as marginal structural models (MSMs; which can account for time-varying variables that act as both confounders and mediators). Regarding alternative designs, twin studies and other family-based designs control automatically for shared confounders, as do negative controls [18]. Natural experiments are another alternative, mimicking the random allocation of RCTs and thus guarding against confounding and reverse causation. These include instrumental variables (IV) designs, where as-if/randomly allocated proxies for exposures are used in place of exposures themselves. Mendelian Randomization (MR), a kind of IV design, offers particular promise given the potential of genetic proxies for alcohol consumption.

While these methods are still limited in their approach to inferring causal relationships from observational data, they represent significant improvements over conventional analyses (see Table 1 for a full list of methods of interest, their advantages, and their limitations). Some of these approaches are gaining popularity [19], but they are not routinely applied to alcohol–long-term health research [20]. Importantly, reviews in this area rarely focus on improved analytical methods to counter confounding and tend to exclude novel study designs. This review therefore aims to identify all observational studies employing such approaches, and to synthesize their findings on the functional form and strength of alcohol–long-term health relationships.

**Table 1:** Methods to enhance causal inference in observational research

Method	Relevant sub-methods	Description	Can address:				Advantages	Limitations
			Confounding	Reverse causality	Selection bias	Measurement error		
**Analytical methods applied to traditional longitudinal study designs**								
Propensity scores (PS) [11, 19]	Covariate balancing propensity scores (CBPS)	-The PS is a single value reflecting the probability of exposure for an individual given their values on all relevant covariates -PS generation occurs as a data ‘pre-processing’ step prior to main analysis -Usually generated via logistic regression -Once generated, the PS can be used for matching, stratification, weighting, or as a covariate for adjustment in regression	✓				-Unlike standard methods, can handle large numbers of covariates -Not reliant on correctly modelling covariate- outcome relationships -Covariate balance after matching/weighting can be assessed	-Still relies on appropriate choice of covariates and accurate measurement -PS matching may not find matches for some treatment cases, leading to reduced sample size and limiting generalizability -Effect estimation with PS doesn’t always perform better than regular adjustment -Most PS methods rely on manual covariate balance checking and refitting
G-methods [21–24]		-A family of methods intended for use with time-dependent variables -Developed as a solution to the problem of time-varying covariates affected by past exposure, including those that act as both confounders and mediators over time -The three G-methods are the G-formula, marginal structural models, and G-estimation, each relying on its own modelling assumptions					-Unlike standard methods to control for confounding, G-methods do not fix values of covariates, thus do not block mediation via the covariate, and avoid introducing collider bias -Accounting for changes in variables over time mitigates misclassification	-Still relies on appropriate choice of covariates and accurate measurement
	G-formula (aka G-computation or G-standardization) [21, 23, 25, 26]	-First models relationship given observed data (using actual exposure for each individual), and then predicts outcomes under counterfactual exposures, with the difference taken as the causal effect -Is a generalization of standardization (conditioning on covariates and then marginalizing) that accounts for dynamic variables by considering covariate distribution over follow-up time	✓		✓ (specifically, bias due to censoring)	✓	-Can be used to calculate risk ratios and risk differences -Well-suited to assess time-varying exposures	-Vulnerable to the ‘g-null paradox’- null hypotheses tend to be rejected in large studies even when true -Usually requires specifying a statistical model, hence also being known as the ‘parametric’ G-formula
	Marginal structural models (MSMs) [21, 23, 25]	-Use weights based on inverse probability of exposure at each time point to create a pseudo-population where each combination of covariates is equally present in each exposure condition -Using these weights, MSMs then estimate the causal effect -The most popular of the G-methods	✓		✓ (specifically, bias due to censoring)	✓	-Simplest of the G-methods to understand and implement -Can also integrate censoring weights to account for differential attrition	-Not ideal for assessing exposure-confounder interactions, with standard MSMs unable to estimate interactions involving dynamic variables -Requires checking weight distribution, may require refitting (as with PS methods) -Cannot be used if all participants are exposed/unexposed on a particular level of a confounder
	G-estimation of structural nested models [23–25]	-At each wave assesses the relationship between exposure and likelihood of outcome given covariates, adjusting for exposure and covariate values from past waves, thus accounting for dynamic confounders affected by past exposure -Considered semi-parametric in that mean counterfactual outcomes under no exposure are unspecified	✓			✓	-Can be used even if all participants are exposed/ unexposed on a particular level of a confounder -Can be used to assess exposure-confounder interactions	-Unlike the other G-methods, cannot account for selection bias arising from censoring, so data requires preliminary weighting to account for bias from censoring
Doubly robust methods [26]	Targeted maximum likelihood estimation; Augmented inverse probability weighting	-Incorporates both an estimation of the outcome mechanism (as in regression adjustment) and the exposure mechanism (as in propensity scores)	✓				-Only the outcome mechanism or the exposure mechanism need be consistently estimated to generate an unbiased estimate of exposure effect	-Still relies on appropriate choice of covariates and accurate measurement
Fixed effects regression [27–31]		-A technique developed in the econometrics literature for use with longitudinal data with repeat outcome measurements, only using information on within-subject variation, thus controlling for all time-invariant sources of confounding -Treats time-invariant characteristics that differ between individuals as fixed parameters (unlike in mixed models), allowing estimation of parameters of interest net of stable confounders -Each participant serves as own control	✓				-Removes the threat of all observed and unobserved time-invariant confounding -Models can be extended to include time-varying covariates	-Cannot overcome time-varying confounding without extending the model, and these variables must be observed/measured -Individuals with stable exposure values do not contribute to estimates; also leads to imprecision when exposures change little over time -Reducing confounding comes at the cost of more sampling variability -Cannot generate parameter estimates for stable characteristics like race
Causal mediation analysis [32–34]		-Integrates traditional mediation analysis (which separately estimates total effect of exposure on outcome, indirect effect via mediators, and direct effect unexplained by mediators) with the potential outcomes framework to allow for exposure-mediator interaction and non-linear relationships (i.e., is a non-parametric method) -Uses the concepts of ‘controlled direct effect’, ‘natural direct effect’, and ‘natural indirect effect’ -Makes explicit underlying assumptions related to unmeasured confounding, and encourages sensitivity analyses to test robustness to assumption violations	✓ (see advantages and limitations*)				-Effect decomposition is still possible given exposure-mediator interaction, nonlinearity, and categorical variables -Makes underlying assumptions explicit -Helps identify the mechanism/s of an exposure’s effect; especially useful when heterogenous causal mechanisms at play -Can be extended to situations with multiple mediators -*If a variable completely mediates the exposure-outcome relationship and is shielded from confounders, confounder measurement is not needed	-*In practice, likely there will always be exposure-mediator or mediator-outcome confounding, so still need to observe and accurately measure covariates -Analyses make strong assumptions, necessitating sensitivity analyses
**Alternative observational study designs**								
Natural experiments [17, 35–37]		-Mimic RCTs by exploiting exogenous events that are truly randomized/approximate random assignment -Differ from true experiments in that exposure is not assigned by the researcher -Assignment may be as a result of naturally occurring phenomena (e.g., a weather event), or of human intervention implemented for reasons other than the research question (e.g., army draft lottery)					-In approximating randomization, obviates the need for accurate measurement of confounders -Potential to overcome measurement error, reverse causation, and selection bias	-Rare to find truly random or as if-random exposure assignment
	Standard natural experiments	-Natural experiments where individuals are as-if/randomly assigned to exposure and control groups	✓	✓	✓	✓		-May be difficult to find a standard natural experiment that maps on well to the actual research question of interest
	Instrumental variable analysis	-Assesses the relationship between an as-if/ randomly assigned *proxy* for the exposure of interest and the outcome -A valid instrumental variable must be associated with the exposure of interest, be independent of confounders of the exposure-outcome relationship, and should affect the outcome only via the exposure	✓	✓	✓	✓	-Useful when the exposure itself is difficult to manipulate or measure	-Difficult to find valid instrumental variables -Potential for weak instrument bias i.e., when the instrument explains a small amount of variance in exposure -Relies on assumption that the instrumental variable is not associated with exposure-outcome confounding
	Genetic instrumental variables	-Kind of instrumental variable analysis using genetic variants as proxies for exposure -The most prominent technique is Mendelian Randomisation	✓	✓	✓	✓	-Genes cannot be confounded by environment, cannot be subject to reverse causality, and are stable over time -Multiple variants can be combined to explain more variance in exposure, mitigating weak instrument bias	-Genetic instrumental variables are proxies for lifelong exposure - this period may be longer than what the research question is interested in -Potential for weak instrument bias, pleiotropy (gene affecting more than one phenotype) and linkage disequilibrium (genes more likely to be inherited together) -Possible population stratification (there may be population subgroups with different distributions of genes)
Quasi-experiments [35]		-Like natural experiments, exploit exogenous events to assess relationships between exposures and outcomes, but lack random or as-if random assignment	✓	✓		✓	-Same as for natural experiments	-Without random assignment, confounding is still possible i.e., cause of exposure may also contribute to outcome
Family-based designs [17, 36]	Twin studies; Sibling comparison	-By comparing genetically related participants discordant for the exposure of interest, accounts for confounding from genetic or shared environmental sources	✓				-Controls for unmeasured/unobservable confounding (for shared covariates) -Comparing monozygotic and dizygotic twins enhances understanding of genetic vs. environmental confounding	-Still need to observe and accurately measure non-shared environmental covariates to control for this kind of confounding -May be difficult to find family members discordant for exposure of interest
Negative controls [18, 36]	Negative control exposures; Negative control outcomes	-Have the same confounding structures as the exposure-outcome relationship of interest, but lack a plausible causal mechanism -If association is greater for the relationship of interest than for the negative control, a causal relationship is likely; if not, suggests confounding/other shared biases responsible -May take the form of a negative control exposure or a negative control outcome	✓		✓ (specifically, immortal time bias)		-Can identify when confounding (or assumed shared bias) is responsible for apparent causal effects -Does not require observation/measurement of covariates	-Relies on assumption of no plausible causal mechanism in the negative control relationship -Relies on assumption that same confounding structure shared by relationship of interest and negative control relationship

## Methods

### Search strategy and study selection

This review’s methods are reported in detail in the study protocol, which was registered with PROSPERO (CRD42020185861) and published [38]. Briefly, searches for peer-reviewed, English-language journal articles and grey literature on MEDLINE, PsycINFO, Embase and Scopus were performed in May 2020 with no limits on publication date. Choice of causal inference methods of interest incorporated expert feedback. Search terms were generated by adapting those from recent reviews and searching keywords/index terms of key eligible papers known to the authors, with iterative refinement. These included controlled vocabulary terms and free text words, and related to: 1) alcohol; 2) levels/patterns of drinking 3) observational, longitudinal studies; 4) analytical approaches to improve causal inference that are used in conjunction with conventional study designs; and 5) design-based approaches to improve causal inference. Groups of terms were combined as follows: 1 and 2 and ((3 and 4) or 5). MEDLINE search terms are provided in Table S1. Additionally, reference lists of eligible, retrieved studies were manually searched.

Only human research was eligible. The exposure of interest was level of alcohol consumption (volume over a given period), or level and pattern of consumption (incorporating frequency/heavy episodic drinking). While studies were eligible regardless of their findings on functional form, their methods must have been capable of detecting non-linearity – were it to be present. For this reason, studies were only eligible if they categorized alcohol consumption, and subsequently performed comparisons between a chosen reference category and the other levels of consumption. This approach does not require assuming a functional form (unlike a single regression using a continuous predictor). Specifically, a non-drinking/light drinking reference was required in addition to at least two other levels of consumption (alternative methods of comparison allowing for the detection of non-linearity were permitted for IV/MR designs). Any long-term health outcome was eligible; studies only reporting on acute/short-term conditions (e.g., injury) were excluded. Eligible studies needed to employ one of the pre-specified approaches to improving causal inference (see Table 1). Studies needed to be longitudinal cohort or case-control designs (excepting IV/MR designs). IV/MR studies must have performed formal IV analysis or otherwise provided estimates in terms of predicted alcohol consumption. Reviews and interventional studies were excluded.

Retrieved titles and abstracts were screened by one reviewer (RV), with a second reviewer (JW) additionally screening a random 25%. Full-text articles were independently assessed by two reviewers (RV and JW). A third reviewer (LM) was consulted regarding unresolved discrepancies.

### Data extraction

Extraction was performed independently by two reviewers (RV and JW) using pre-piloted forms. Extracted data included publication details (author/s, year), participant characteristics (sample size, setting, mean age, eligibility criteria, cohort name), exposure details (number and spread of measurement occasions, nature of discretized categories), study design and analysis methods, health outcome/s (how assessed, whether binary/continuous, interval to measurement), and results (relationship strength and form). Study authors were contacted if further information was required (see Table S2).

### Quality assessment

Given the range of designs targeted by this review, two risk of bias assessment tools were used. Cohort studies were assessed using the relevant Newcastle-Ottawa Scale (NOS) [39], and a recently developed tool specific to MR [40] was employed. One reviewer (RV) applied the tools to all studies, with a second reviewer (JW) additionally assessing a random 25%. In line with other similar reviews, formal assessment of evidence quality was limited to risk of bias [40, 41].

### Synthesis and reporting

Data synthesis was limited to narrative description given the heterogeneity in health outcomes and methods employed by included studies. Reporting of this review complies with the Preferred Reporting Items for Systematic Review and Meta-Analysis (PRISMA) [42], the checklist for which can be found in Table S3.

## Results

### Characteristics of included studies

**Fig. 2:**
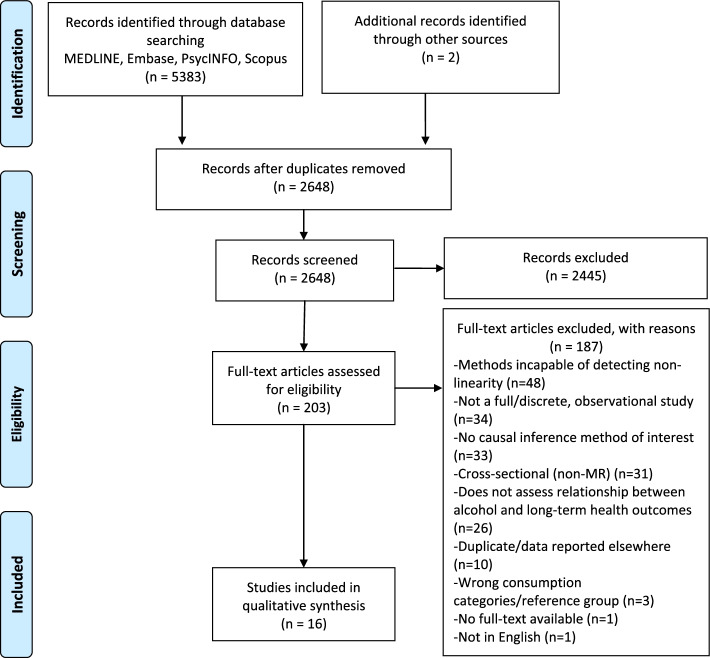
PRISMA flow chart

Sixteen articles met inclusion criteria (see Fig. 2), comprising four MR studies, nine twin designs, and three prospective cohort studies employing MSMs, reporting on health outcomes broadly related to cancer, diabetes, dementia, mental health, cardiovascular health, mortality, HIV, and musculoskeletal health. Two cohorts provided all twin study data, with all but one non-MR study conducted with Swedish or Finnish populations. Study characteristics are summarized in Table 2, and exclusion reasons for key ineligible papers are provided in Table S4. The two reviewers were in agreement on title and abstract screening for 93.13% of cases, and all discrepancies at full-text screening were resolved by discussion between reviewers.

**Table 2:** Characteristics of included papers (*n* = 16)

First author (year)	Health outcome/s and interval to follow-up	Study design	Participant characteristics	Cohort/s name	Sample size	Statistical methodology (reference group bolded)	Explicit testing for non-linearity?	Main results (statistically significant findings bolded)	Conclusion
**Cancer**									
Dickerman (2016) [43]	Prostate cancer (PC) and prostate cancer mortality -median 30 yrs. of follow-up	Twin	Male twins; mean age 40.1 at baseline	Older Finnish Twin Cohort	11,372; 225 and 43 discordant twin pairs for PC and PC-mortality respectively	-Co-twin (discordant for both alcohol consumption level and either time to diagnosis among outcome-concordant pairs concordant or time to event vs death/end of follow-up among outcome-discordant pairs) and pooled cohort Cox analyses to examine risk for PC and PC-mortality -Alcohol consumption measured twice (6 years apart) and averaged; categories: abstainers, **light** (0.01–3 drinks/wk), moderate (> 3–14 drinks/wk), heavy (> 14 drinks/wk); 1 drink considered = 12 g of alcohol	ϰ	-PC-risk: HR (95% CI) Cohort MZ twins DZ twins All twins Abstainers 1.27 (.94,1.71) 2.85 (.67,12.1) **3.80** (1.36,20.6) **2.98** (1.35,6.60) Moderate 1.2 (.99,1.46) 1.28 (.6,2.74) 1.54 (0.92,2.57) 1.36 (.91,2.04) Heavy **1.46** (1.12,1.91) 2.00(.62,6.45) 1.71 (.87,3.39) 1.63 (.92,2.88) -PC-mortality: HR (95% CI) Cohort MZ twins DZ twins All twins Abstainers **1.90** (1.04,3.47) 2.31 (.19, 27.4) 1.83 (.42,8.03) 1.37 (.44,4.28) Moderate 1.22 (.76,1.97) 9.13 (.70,119) 1.43 (.37,5.62) 2.44 (.79, 7.52) Heavy 1.32 (.66,2.62) - 2.39 (.33,17.3) 7.31 (1.3, 41)	**Reverse J-shaped relationships, with light drinking at the nadir, were evident in all twin analyses of PC risk. J-shaped curves were evident in twin analyses of PC mortality. The evidence is consistent with a causal protective effect of light drinking, but twin analyses in general lacked power.**
**Diabetes**									
Carlsson (2003) [44]	Type 2 diabetes (T2D) −20 yrs. of follow-up	Twin	Same-sex twins without diabetes at baseline; mean age 34.3 (M) and 35.4 (F) at baseline	Finnish Twin Cohort	22,788; 27 discordant twin pairs analysed	-Co-twin (discordant for alcohol category) and pooled cohort Cox analyses to examine risk for T2D -Categories for pooled cohort (based on 3 questionnaires over 15 years): abstainers, **< 5 g/day**, 5–19.9 g/day (women)/5–29.9 g/day (men), ≥ 20 g/day (women)/ ≥ 30 g/day (men); for twin analyses (based on baseline questionnaire only): **low** (< 5 g/day), moderate (15–29.9 g/day), high (≥ 30 g/day)	✓	RR (95% CI) Cohort Men Cohort Women OR (95% CI) Twin Abstainers 1.1 (.7,1.5) 1.1 (.9,1.5) Moderate .5 (.2,1.3) 5–29.9 g/day (m); 5–19.9 g/day (f) .8 (.6,1.1) .7 (.4,1.1) High 1.2 (.4,3.9) ≥ 30 g/day (m); ≥ 20 g/day (f) .9 (.6,1.4) 1.6 (.8,3.5)	**Twin analyses were critically underpowered, preventing causal inference.**
Peng (2019) [45] *See also cardiovascular outcomes	Diabetes-related biomarkers (FBG, P2hBG, HbA1c, HOMA-IR, HOMA-beta) -cross-sectional	MR	Chinese adults living in the Yi-Ling district of Yichang; mean age 55 (SD 0.1) at baseline	One community from the Risk Evaluation of cAncers in Chinese diabeTic Individuals: a LONgitudinal (REACTION) study	4536	−1-sample MR using a single variant and standard IV analysis (2SLS); local average treatment effects (LATEs) computed for subgroups of observed alcohol consumption (non-zero LATE slopes indicate non-linearity) -Only standard, linear IV analysis was performed for categorical diabetes risk, not allowing for detection of non-linearity -Analysis performed in women as a negative control due to their lack of alcohol consumption -No conventional analyses conducted for comparison	✓	-Non-linear analyses: the LATE slopes for all diabetes-related biomarkers were not sig. Different to zero, indicating no non-linear relationships Unstandardizedβ (95% CI) Using log-transformed alcohol intake FBG (log-transformed) -.01 (−.04,.01) P2hBG (log-transformed) .01 (−.04,.06) HbA1c (log-transformed) -.01 (−.03,.00) HOMA-IR (log-transformed) -.00 (−.09,.08) HOMA-beta (log-transformed) .03 (−.04, .11) -Standard IV analyses: Unstandardizedβ (95% CI)Per 1-unit increase in log-transformed genetically-predicted alcohol consumption FBG (log-transformed)**.04** (.02,.05) P2hBG(log-transformed)**.07** (.04,.11) HbA1c (log-transformed).00 (−.01,.01) HOMA-IR(log-transformed)**.10** (.04, .17) HOMA-beta (log-transformed).00 (−.05,.06)	**No evidence of non-linear relationships between genetically predicted alcohol consumption and diabetes-related biomarkers. Alcohol appears to raise FBG, P2hBG and HOMA-IR in a linear fashion in males, although these are small effects.**
**Dementia**									
Handing (2015) [46]	Dementia −43 yrs. of follow-up	Twin	Twins without dementia prior to baseline; <=65 at baseline and > =60 at study end; mean age 54.2 (SD 5.9) at baseline; reported drinking <=100 g/day of alcohol	Swedish Twin Registry	12,362; 576 dementia discordant pairs (177 MZ); 396 concordant pairs (160 MZ)	-Co-twin (discordant for dementia) logistic regressions and pooled cohort Cox models used to examine risk for dementia; co-twin (concordant for dementia) mixed-effects analyses used to examine age at onset -Categories for pooled cohort + twin age of onset analyses: abstainers, **light** (> 0 and ≤ 5 g/d), moderate (> 5 and ≤ 12 g/d), heavy (> 12 and ≤ 24 g/d), and very heavy (> 24 g/d). For twin dementia risk analyses: abstainers, **light** (> 0 and ≤ 5 g/d), and moderate-to-very heavy (> 5 g/d)	✓	-Dementia risk: HR (95% CI) Cohort OR (95% CI) MZ twins All twins Abstainers 1.05 (1.00,1.11) Abstainers 1.37 (.6,3.16) 1.39 (.89,2.16) Moderate .98 (.92–1.04) Moderate-to-very heavy **3.07** (1.37,6.86) **1.57** (1.04,2.37) Heavy **1.1** (1.01,1.19) Very heavy **1.18** (1.01,1.36) -Dementia age at onset: Mean difference in years between twin diagnosed first vs twin diagnosed later (*p* value) MZ twins All twins Abstainers − 5.37 (.83) -5.49 (.68) Light −6.28 (NA – reference group) -6.79 (NA – reference group) Moderate − 5.41 (.13) -7.00 (.98) Heavy − 6.33 (.99) -6.73 (.32) Very heavy − 12.67 (.09) **-10.67** (.02)	**Results are consistent with a J-shaped curve. Twin analyses do support a causal role for increased risk of dementia for moderate-to-very heavy drinking, and faster dementia onset for very heavy drinkers.**
**Mental health**									
Gemes (2019) [47]	Depression − 7-9 yrs. of follow-up	MSM	General population aged 20–64 at recruitment; mean age 43.3 (SD 12.2) at baseline	Psykisk Hälsa–Arbete–Relationer (PART) cohort (Sweden)	5087	-MSM (weighted logistic regression) + standard logistic regression for comparison -Alcohol consumption measured pre-baseline (for use as time-variant confounder) and baseline; categories: no consumption, **light** (> 0- ≤ 7 drinks/wk), moderate (> 7- ≤ 14 drinks/wk), and excessive (> 14 drinks/wk); 1 drink = 12 g	✓	-Adjusting for baseline MDI score: RR (95% CI) Non-MSM MSM Abstainers 1.10 (.69,1.74) **1.60** (1.27,2.01) Moderate .54 (.28,1.04) 1.05 (.83,1.32) Excessive .61 (.21,3.07) **1.77** (1.13,2.78) -Excluding those with baseline depression (MDI > 26): RR (95% CI) Non-MSM MSM Abstainers 1.24 (.72,2.14) **1.46** (1.10,1.95) Moderate .63 (.29,1.41) .75 (.55,1.03) Excessive 2.72 (.61,12.19) **2.83** (1.80,4.44)	**MSM results support a non-linear (U/J-shaped) relationship between alcohol consumption and depression.**
Samuelsson 2013 [48] ^a^	Disability pension (DP) due to mental health diagnoses (MHD) -median 10 yrs. of follow-up from prior study (baseline)	Twin	Twins with data from a prior study, and at time of that study were living in Sweden, < 65, and without DP/old age pension; mean age 52.9 (SD 5.6)	Swedish Twin Study of Disability Pension and Sickness Absence (STODS), drawn from the Swedish Twin Registry	28,613; 229 DP-discordant twin pairs (95 MZ pairs)	-Co-twin (discordant for dementia) Cox regressions + pooled cohort Cox regressions performed -Differentiated between frequent and infrequent drinkers (have/not consumed alcohol in previous two months); categories for pooled cohort + twin analyses: abstainers, **light frequent** (12-84 g/wk), moderate frequent (M: 85-168 g/wk.; F: 85–108 g/wk) and ≤ 12 g/d), heavy frequent (M: > 168 g/wk.; F: > 108 g/wk), light infrequent (12 g/occasion), moderate infrequent (M:13-48 g/occasion; F: 13-36 g/occasion), heavy infrequent (M: > 48 g/occasion; F:> 36 g/occasion)	✓	HR (95% CI) Cohort MZ twins DZ twins All twins Abstainers **1.99** (1.57,2.54) 1.93 (.54,6.96) **2.63** (1.05,6.62) **2.17** (1.06,4.45) Moderate frequent 1.07 (.78,1.49) .46 (.11,1.87) 2.70 (.81–9.02) 1.24 (.53,2.91) Heavy frequent .98 (.61,1.54) - .46 (.09,2.36) .48 (.12,1.90) Light infrequent 1.09 (.69,1.73) 2.80 (.24,32.6) 3.98 (.71,22.4) 3.67 (.91,14.8) Moderate infrequent 1.18 (.91,1.54) .52 (.18,1.49) 1.71 (.75,3.91) 1.03 (.55,1.93) Heavy infrequent 1.20 (.92,1.57) 1.09 (.33,3.53) **3.61** (1.28,10.2) 2.10 (.99,4.46)	**Results support a non-linear relationship between alcohol consumption and DP due to MHD, such that abstainers are at increased risk compared to light frequent drinkers. Light and heavy infrequent drinkers are also at increased risk, but CIs are very wide for the former, and effect disappears for the latter in MZ-only twins.**
**Cardiovascular events/diagnoses**									
Ilomaki (2011) [49]	Myocardial infarction (MI) − 12-14 yrs. of follow-up	MSM	General population males; mean age 52 (SD 6.7) at initial exam (4 yrs. before ‘baseline’)	Kuopio Ischaemic Heart Disease Risk Factor Study (KIHD); Finland	1030	−5 discrete-time hazard models run and compared: M1 (baseline alcohol consumption with no covariate adjustment or inclusion of t-4 consumption), M2 (baseline alcohol consumption adjusted for covariates and t-4 consumption), M3 (baseline and t + 7 alcohol consumption with no covariate adjustment or inclusion of t-4 consumption), M4 (baseline and t + 7 alcohol consumption adjusted for covariates and t-4 consumption), M5 (MSM with baseline and t + 7 alcohol consumption with stabilized IP weights at t and t + 7) -Baseline alcohol categories: < 12 g/wk., **12-83 g/wk**, 84-167 g/wk., ≥168 g/wk.; additional alcohol measurements at t-4 and t + 7 included in various models	✓	RR (95% CI) M1 M2 M3 M4 M5 (MSM) < 12 g/wk. 1.20 (.86,1.67) 1.01 (.70,1.45) **1.55** (1.08,2.21) 1.27 (.88,1.81) 1.27 (.88,1.83) 84-167 g/wk. 1.05 (.71,1.56) 1.13 (.75,1.72) 1.20 (.78,1.84) 1.27 (.81,2.00) 1.18 (.75,1.87) ≥168 g/wk. .98 (.60,1.58) 1.20 (.68,2.12) 1.40 (.91,2.18) **1.71** (1.03,2.85) 1.59 (.93,2.72)	**Results are generally consistent with increased risk for MI for those drinking less than weekly and those drinking heavily. However, effect sizes varied with model specifications.**
Kadlecova (2015) [50]	Stroke and transient ischaemic attack (TIA) events − 43 yrs. of follow-up	Twin	Same-sex twins ≤60 with no history of stroke at baseline and with ≥5 yrs. of follow-up; mean age 50.5 (SD 5.29) at baseline	Swedish Twin Registry	11,644; 370 stroke/TIA discordant pairs (all MZ); 167 stroke/TIA concordant pairs (all MZ)	-Co-twin (discordant for stroke) logistic regressions and pooled cohort Cox models used to examine risk for stroke/TIA; co-twin (concordant for stroke) mixed-effects analyses used to examine time to stroke/TIA -Categories for pooled cohort + twin analyses: abstainers, **very light** (> 0-5 g/day), light (> 5 − 12 g/day), moderate (> 12-24 g/day), heavy (> 24 g/day)	✓	-Stroke/TIA risk: HR (95% CI) Cohort OR (*p* value) Twins Abstainers 1.11 (.98,1.23) Abstainers 2.22 (.058) Light .98 (.85,1.15) Light 1.56 (.17) Moderate .99 (.85,1.15) Moderate 1.59 (.26) Heavy **1.34** (1.04,1.70) Heavy 1.23 (.78) -Time to stroke/TIA: Mean difference in yrs. to stroke/TIA (*p* value) Twins Abstainers 2.07 (.11) Light .77 (.54) Moderate 1.15 (.47) Heavy **− 5.68** (.029)	**Twin analyses are consistent with an increased risk of stroke/TIA for abstainers compared to very light drinkers, with somewhat increased risks for heavier drinking groups as well (reverse J-shape). Results support a causal role of heavy alcohol consumption in hastening time to event among those who experience stroke/TIA.**
Millwood 2019 [51]	Ischaemic stroke, intracerebral haemorrhage (ICH), total stroke, acute myocardial infarction (AMI), total coronary heart disease (CHD) -roughly 10 yrs. of follow-up	MR	Permanent residents from 10 Chinese regions aged roughly 35–74 and without major disabilities, (those with a history of CVD were excluded from analyses of disease incidence); mean age 52 (SD 11) at baseline	China Kadoorie Biobank	512,715; 161,498 of which had genotype data (male and female combined)	-1-sample MR using Cox models -Instrument composed of 9 combinations of 2 SNPs (ALDH2 rs671 and ADH1B rs1229984) within each of 10 geographic areas, producing 90 combinations overall; then, based on mean ‘usual’ alcohol consumption within each of those combinations (incorporating repeat measurements to account for measurement error), six final categories were produced, aligned with increasing genetically-predicted alcohol consumption: **Category1: 0-10 g/wk**, Category2: 10-25 g/wk., Category 3: 25-50 g/wk., Category 4: 50-100 g/wk., Category 5100-150 g/wk., Category 6 > 150 g/wk. -Conventional analyses using observed alcohol consumption conducted for comparison using Cox models, with consumption categories (for men): ex-drinker, non-drinker, occasional drinker (less than weekly), **< 140 g/wk**, 140-279 g/wk., 280-419 g/wk., ≥420 g/wk	✓	-Ischaemic stroke: Log RR (95% CI) MR Conventional C2 1.00 (.91,1.10) Ex-drinker **1.39** (1.32,1.46) C3 1.03 (.96,1.11) Non-drinker **1.21** (1.16,1.25) C4 **1.11** (1.02,1.20) Occasional 1.00 (.97,1.03) C5 **1.23** (1.15,1.33) 140-279 g/wk. **1.13** (1.07,1.19) C6 **1.23** (1.12,1.35) 280-419 g/wk. **1.23** (1.15, 1.32) ≥420 g/wk. **1.31** (1.21,1.41) Per 280 g/wk. (assuming linearity) **1.27** (1.13,1.43) **1.28** (1.19,1.38) -ICH: Log RR (95% CI) MR Conventional C2 1.01 (.88,1.14) Ex-drinker **1.52** (1.38, 1.68) C3 1.02 (.88,1.14) Non-drinker **1.33** (1.25,1.43) C4 1.08 (.96,1.22) Occasional 1.02 (.96,1.09) C5 **1.29** (1.15,1.44) 140-279 g/wk. **1.35** (1.21,1.52) C6 **1.54** (1.36,1.76) 280-419 g/wk. **1.52** (1.32,1.74) ≥420 g/wk. **1.73** (1.52,1.97) Per 280 g/wk. (assuming linearity) **1.58** (1.36,1.84) **1.59** (1.37,1.85) -Total stroke: Log RR (95% CI) MR Conventional C2 1.02 (.95,1.10) Ex-drinker **1.40** (1.34,1.46) C3 1.05 (.95,1.10) Non-drinker **1.23** (1.19,1.27) C4 **1.13** (1.06,2.10) Occasional 1.00 (.98, 1.03) C5 **1.27** (1.19,1.34) 140-279 g/wk. **1.16** (1.10,1.21) C6 **1.35** (1.26,1.45) 280-419 g/wk. **1.28** (1.20,1.36) ≥420 g/wk. **1.39** (1.31,1.48) Per 280 g/wk. (assuming linearity) **1.38** (1.26,1.51) **1.35** (1.27,1.44) -AMI: Log RR (95% CI) MR Conventional C2 1.02 (.87,1.19) Ex-drinker **1.66** (1.48,1.85) C3 1.05 (.93,1.19) Non-drinker **1.63** (1.51,1.76) C4 .93 (.81,1.07) Occasional **1.23** (1.16,1.31) C5 .94 9.81,1.09) 140-279 g/wk 1.11 (.97,1.26) C6 .97 (.83,1.15) 280-419 g/wk **1.19** (1.01,1.41) ≥420 g/wk 1.14 (.95,1.37) -Total CHD: Log RR (95% CI) MR Conventional C2 1.03 (.93,1.13) Ex-drinker **1.45** (1.38,1.52) C3 **1.08** (1.01,1.15) Non-drinker **1.31** (1.26,1.36) C4 .94 (.87,1.02) Occasional **1.07** (1.04,1.11) C5 1.04 (.97,1.11) 140-279 g/wk 1.03 (.97,1.09) C6 1.06 (.98,1.15) 280-419 g/wk **1.11** (1.03,1.19) ≥420 g/wk **1.13** (1.04,1.22)	**In contrast to conventional analyses, MR results are consistent with a monotonically increasing relationship between alcohol and stroke events. The results do not support a causal relationship between alcohol consumption and AMI or total CHD.**
Ropponen 2014 [52] *See also musculoskeletal health	DP due to circulatory system diagnoses −5 − 10 years of follow-up	Twin	Twins with data from a prior study, and at time of that study were living in Sweden, < 65, working and without DP/old age pension; mean age at baseline 53.7 (SD 5.7) ^a^	Swedish Twin Registry	31,206; 216 DP due to circulatory system diagnoses-discordant pairs (of which 95 are MZ)	-Co-twin (discordant for DP due to MSD) Cox models and pooled cohort Cox models used to examine risk (stratified for sex in pooled model) -Categories for pooled cohort + twin analyses: abstainers, **light** (≤3 drinks/wk), moderate (F: > 3- ≤ 7 drinks/wk.; M: > 3- ≤ 14 drinks/wk), heavy (F: >7drinks/wk.; M: > 14 drinks/wk)	✓	HR (95% CI) Cohort MZ twins DZ twins All twins Abstainers 1.22 (.91,1.64) .97 (.31,3.01) 1.55 (.70,3.44) 1.3 (.69,2.45) Moderate .85 (.63,1.15) - .86 (.39,1.91) 1.54 (.78,3.04) Heavy **.79** (.63,.98) .91 (.49,1.68) .89 (.52,1.51) .86 (.57,1.28)	**Results do not offer clear support for causal relationships between alcohol consumption and later receipt of DP due to circulatory system diagnoses.**
**Cardiovascular disease biomarkers**									
Peng (2019) 28 *See also diabetic outcomes	Lipids: HDL-C, non-HDL-C, triglycerides (TG), total cholesterol (TC); blood pressure: systolic blood pressure (SBP), diastolic blood pressure (DBP); obesity anthropometric measures: BMI, waist circumference (WC), hip circumference (HC), waist-to-hip ratio (WHR) -cross-sectional	MR	Chinese adults living in the Yi-Ling district of Yichang; mean age 55 (SD 0.1) at baseline	One community selected from the Risk Evaluation of cAncers in Chinese diabeTic Individuals: a LONgitudinal (REACTION) study	4536	−1-sample MR using ALDH2 to instrument for alcohol consumption with standard IV analysis (2SLS); local average treatment effects (LATEs) computed for subgroups of observed alcohol consumption (non-zero LATE slopes indicate non-linearity) -Analysis performed in women as a negative control due to their lack of alcohol consumption -No conventional analyses conducted for comparison	✓	-Non-linear analyses: the LATE slopes for all lipids, blood pressure and obesity parameters in both sexes were not sig. Different to zero, indicating no presence of non-linear relationships Unstandardizedβ (95% CI) Using log-transformed alcohol intake HDL-C −.01 (−.06,.04) Non-HDL-C −.01 (−.13,.10) TG (log-transformed) .03 (−.05,.10) TC −.03 (−.16,.08) SBP −.85 (− 3.15,1.3) DBP −.70 (− 2.32,.71) BMI −.15 (−.51,.21) WC −.02 (− 1.09,1.09) HC .34 (−.37,1.07) WHR −.00 (−.01,.00) -Standard IV analyses: Unstandardizedβ (95% CI) Per 1-unit increase in log-transformed genetically-predicted alcohol consumption HDL-C .04 (−.00,.08) Non-HDL-C **.11** (.02,.20) TG (log-transformed) **.11** (.05,.16) TC **.15** (.06,.25) SBP **2.91** (1.06,4.76) DBP **3.03** (1.87,4.19) BMI **.57** (.28,.87) WC **2.37** (1.47,3.27) HC **1.01** (.39,1.62) WHR .02 (.00,.02)	**LATE results offer no evidence of non-linear relationships between genetically predicted alcohol consumption and lipid markers, blood pressure or obesity parameters. Alcohol appears to raise BMI, WC, HC, non-HDL-C, TG, TC, SBP and DBP in a linear fashion in males, with little effect on HDL-C or WHR.**
Silverwood (2014) [53]	Lipids: non-HDL-C, HDL-C, TG; blood pressure: SBP; obesity anthropometric measures: BMI, WC; inflammatory markers: CRP, interleukin 6 (IL-6) -cross-sectional	MR	Individuals of European descent from Europe and North America; mean age 56.75 (calculated from Holmes et al. [cite])	22 individual studies (18 cohorts, 2 nested case-control, 1 RCT, 1 case-control) from the Alcohol-ADH1B consortium	80,057 individuals total; 78,172 for SBP, 60,140 for non-HDL-C, 60,227 for HDL-C, 79,454 for BMI, 57,172 for WC, 63,367 for CRP, 23,535 for IL-6, 63,667 for TG	-1-sample MR using the rs1229984 polymorphism in ADH1B to instrument for alcohol consumption and standard IV analysis (2SLS); local average treatment effects (LATEs) computed for subgroups of observed alcohol consumption (non-zero LATE slopes indicate non-linearity) -Where non-linearity is present, the difference in outcome between no alcohol and median observed consumption in low (> 0–7 units/wk), moderate (7–21 units/wk), heavy (21–70 units/wk) and very heavy (> 70 units/wk) groups is predicted, as well as curve nadir, difference in outcome between nadir and abstinence, and level of consumption matching outcome for abstinence -No conventional analyses conducted for comparison	✓	-Non-linear analyses: the LATE slopes for SBP, non-HDL-C, BMI, WC and CRP were all sig. Different to zero, indicating non-linearity Unstandardizedβ (95% CI) Using log-transformed alcohol intake HDL-C .00 (−.06,.06) Non-HDL-C **.37** (.19,.55) TG (log-transformed) -.02 (−.10,.06) SBP **3.30** (1.0,5.5) BMI **.90** (.3,1.4) WC **2.00** (.6,3.6) CRP (log-transformed) **.26** (.10,.43) IL-6 (log-transformed) -.13 (−.34,.29) -Predicted differences in outcome between category medians and abstinence (unstandardized): 3.04 units/wk. 12.15 units/wk. 31.90 units/wk. 84.52 units/wk. Non-HDL-C −.39 (−.79,.06) -.15 (−.72,.47) .40 (−.28,1.10) **1.30** (.45,2.16) SBP .1 (− 5.5,6.1) 5.2 (− 2.6,13.9) **12.4** (3.4,22.1) **22.8** (12.2,34.6) BMI −.6 (− 2.2,.8) .2 (− 2.0,2.1) 1.6 (−.8,3.8) **3.9** (1.2,6.3) WC −.6 (− 4.7,3.5) 1.9 (− 3.9,7.8) 5.7(−.6,12.5) **11.5** (4.5,12.5) CRP (log-transformed) -.29 (−.68,.15) -.15 (−.68,.5) .22 (−.37,.95) **.83** (.15,1.69) -Predicted curve features for those outcomes with evidence of non-linearity (unstandardized): Nadir (units/wk.; 95% CI) Difference in outcome at nadir Units/wk. (95% CI) with outcome equivalent to abstinence Non-HDL-C **3.2 (**.7,6.0) **−.39** (−.85,-.03) **16.9** (2.1, 48.2) SBP 1.00 (.0,3.6) −.7(− 5.4,.0) 2.8 (.0,19.6) BMI 2.3 (.0,6.0) −.6 (− 2.3,.0) 10.1 (.0,48.4) WC 1.5 (.0,5.4) −.8 (− 4.9,.0) 5.3 (.0,37.4) CRP 3.5 (.0,7.2) −.30 (−.75,.00) 19.4 (.0,66.0) -Standard IV analyses for those outcomes with no evidence of non-linearity: β (95% CI) Per 1-unit increase in log-transformed genetically-predicted alcohol consumption HDL-C −.02 (−.07,.03) TG (log-transformed) −.01 (−.06,.07) IL-6 (log-transformed) **.30** (.16,.45)	**Results support non-linear relationships between alcohol consumption and SBP, non-HDL-C, BMI, WC and CRP, with nadirs for these outcomes falling in the low drinking range. These relationships are best characterised by J-shapes with shallow nadirs, followed by gentle, elongated inclines. Results are consistent with a positive linear relationship between alcohol and IL-6, and do not support any causal relationships between alcohol and HDL-C or TG.**
Vu (2016) [54]	Lipids: TG, total cholesterol, HDL-C, HDL2-C, HDL3-C, LDL-C, sdLDL-C, apoB, Lp(a) -cross-sectional	MR	European Americans; mean age 54.3 (SD 5.7) at baseline	Atherosclerosis Risk in Communities (ARIC); USA	10,893 individuals total; 9911 for TG, 9751 for total cholesterol and LDL-C, 10,132 for HDL-C, 10,120 for HDL2-C and HDL3-C, 8102 for sdLDL-C, 7663 for apoB, 9924 for Lp(a)	− 1-sample MR using a genetic risk score composed of 5 SNPs (rs2066702, rs1693457, rs1789891, rs698, and rs1126671) and standard IV analysis (2SLS); model fitted separately for quartiles of genetically-predicted alcohol consumption (**q1 = 1.49–3.63 g/wk,** q2 = 3.63–4.66 g/wk., q3 = 4.66–10.57 g/wk., q4 = 10.57–19.54 g/wk) to determine presence of non-linearity -Conventional analyses regressing outcomes on observed alcohol consumption were also conducted, using consumption categories: **never drinkers**, former/infrequent drinkers (< 1 drink/wk), low-to-moderate current drinkers (M: ≤210 g/wk., F: ≤105 g/wk), heavy current drinkers (M: > 210 g/wk., F: > 105 g/wk)	✓	-TG (log-transformed): Unstandardized β (95% CI) MR Using log-transformed alcohol intake Conventional Q2–**.06** (−.09,-.02) Former/ infrequent **−.08** (−.11,-.05) Q3–**.13** (−.20,-.07) Low-to-moderate **−.16** (−.19,-.13) Q4–**.08** (−.17,.00) Heavy **−.13** (−.17,-.09) -TC: Unstandardized β (95% CI) MR Using log-transformed alcohol intake Conventional Q2–**5.54** (− 8.23,-2.85) Former/ infrequent **− 2.75** (− 4.99,-.51) Q3–**7.71** (− 13.26,-2.15) Low-to-moderate −.73 (− 3.03,1.57) Q4–4.56 (− 11.36,2.25) Heavy **4.05** (.73,7.37) -HDL-C (log-transformed): Unstandardized β (95% CI) MR Using log-transformed alcohol intake Conventional Q2 .01 (−.01,.03) Former/ infrequent **.03** (.01,.04) Q3 .04 (.00,.07) Low-to-moderate **.14** (.12,.15) Q4 .03 (−.02,.07) Heavy **.26** (.23,.28) -HDL2-C (log-transformed): Unstandardized β (95% CI) MR Using log-transformed alcohol intake Conventional Q2 **.04** (.00,.07) Former/ infrequent **.07** (.04,.10) Q3 **.10** (.03,.17) Low-to-moderate **.17** (.14,.20) Q4 .06 (−.03,.15) Heavy **.30** (.26,.35) -HDL3-C: Unstandardized β (95% CI) MR Conventional Q2–.19(−.87,.49) Former/ infrequent .40 (−.14,.94) Q3 .08 (− 1.23,1.38) Low-to-moderate **4.16** (3.60,4.73) Q4 .11 (− 1.51,1.74) Heavy **8.70** (7.84,9.55) -LDL-C: Unstandardized β (95% CI) MR Using log-transformed alcohol intake Conventional Q2 **−4.60** (−7.18,-2.03) Former/ infrequent **−2.59** (− 4.69,-.49) Q3 **−6.87** (− 12.24,-1.50) Low-to-moderate **− 4.38** (− 6.53,-2.23) Q4 − 4.57 (− 11.11,1.96) Heavy **−7.48** (− 10.70,-4.25) -sdLDL-C (log-transformed): Unstandardized β (95% CI) MR Using log-transformed alcohol intake Conventional Q2 **−.04** (−.08,-.01) Former/ infrequent **−.04** (−.07,-.01) Q3 **−.08** (−.15,-.01) Low-to-moderate **−.05** (−.08,-.01) Q4 −.08 (−.17,.01) Heavy .01 (−.04,.06) -apoB (log-transformed): Unstandardized β (95% CI) MR Using log-transformed alcohol intake Conventional Q2 **−.03** (−.04,−.01) Former/ infrequent -.01 (−.02,.01) Q3 −.04 (−.07,.00) Low-to-moderate −.01 (−.03,.01) Q4 −.04 (−.08,.01) Heavy −.02 (−.04,.01) -Lp(a) (log-transformed): Unstandardized β (95% CI) MR Using log-transformed alcohol intake Conventional Q2 −.02 (−.09,.06) Former/ infrequent −.03 (−.10,.03) Q3 −.05 (−.20,.10) Low-to-moderate .01 (−.06,.08) Q4 −.01 (−.20,.18) Heavy −.06 (−.16,.03)	**Results support causal relationships between alcohol and TG, TC, HDL2-C, LDL-C, sdlDL-C and apoB, such that some level of consumption leads to more favorable levels than abstinence/very low consumption does. Analyses suggest non-linearity, with benefits peaking for most outcomes at roughly .5–.1 drinks per week, although effect sizes vary. Results do not support causal relationships between alcohol and HDL-C, HDL3-C and Lp(a).**
**Mortality**									
Sipila 2016 [55]	All-cause mortality -median 30.2 yrs. of follow-up	Twin	Same-sex twins aged 18–54 at baseline and free of chronic disease 6 years post-baseline; mean age 35.9 at baseline	Older Finnish Twin Cohort	14,787; 3389 drinking-discordant pairs (of which 926 pairs are MZ) ^a^	-Co-twin (discordant for alcohol consumption) Cox models and pooled cohort Cox models used to examine risk for mortality -Categories for pooled cohort + twin analyses based on average of two measurements 6 yrs. apart: abstainers, **1-69 g/mnth**, 70-139 g/mnth, 140-209 g/mnth, 210-419 g/mnth, 420-839 g/mnth, 840-1199 g/mnth, ≥1200 g/mnth	ϰ	HR (95% CI) Cohort MZ twins All twins 0 g/mnth 1.02 (.85,1.22) .43 (.17,1.11) .96 (.63,1.45) 70–139 g/mnth .95 (.81,1.10) .64 (.29,1.40) .96 (.68,1.36) 140–209 g/mnth 1.08 (.91,1.29) 1.12 (.47,2.65) .85 (.57,1.26) 210–419 g/mth **1.29** (1.09,1.53) 1.55 (.65,3.71) 1.40 (.94,2.09) 420–839 g/mnth **1.56** (1.31,1.85) 1.70 (.69,4.22) **1.60** (1.06,2.43) 840–1199 g/mnth **2.17** (1.74,2.70) 1.65 (.54,5.08) **2.65** (1.50,4.69) ≥1200 g/mnth **2.81** (2.26,3.50) 3.18 (.81,12.43) **2.99** (1.60,5.59)	**Results are consistent with a monotonically increasing risk function. Twin analyses support a causal role for increased risk of all-cause mortality for moderate-to-very heavy drinking.**
**HIV seroconversion**									
Sander (2013) [56]	HIV seroconversion -median 10.5 yrs. of follow-up	MSM	Men who have sex with men and who were sexually active and HIV-seronegative at baseline; median age 33.4 at baseline	Multicenter AIDS Cohort Study (MACS); USA	3752	-MSM (Cox models) + standard Cox models for comparison -Categories for analyses based on average of two measurements 1 yr apart: **abstainers**, moderate (1–14 drinks/wk), heavy (> 14 drinks/wk); 1 drink considered = 14 mL	ϰ	RR (95% CI) Non-MSM MSM Moderate .91 (.65,1.27) 1.10 (.78,1.54) Heavy 1.19 (.83,1.70) **1.61** (1.12,2.29)	**Results are consistent with a monotonically increasing risk function. Abstainers and moderate drinkers appear to have similar risk for HIV seroconversion, while heavy drinkers are at increased risk.**
**Musculoskeletal health**									
Pietikainen 2011 [57]	DP due to low back disorders (LBD) − 29 years of follow-up	Twin	Same-sex twins aged 18–64 and not receiving pension at baseline; mean age 33.2 (SD 12) at baseline	Finnish Twin Cohort	24,043; 504 (284 M) pairs discordant for DP due to LBD	-Co-twin (discordant for DP due to LBD) Cox models and pooled cohort Cox models used to examine risk for mortality -Categories for pooled cohort + twin analyses: abstainers, l**ight (≤3 drinks/wk),** moderate (F: > 3- ≤ 7 drinks/wk.; M: > 3- ≤ 14 drinks/wk), heavy (F: >7drinks/wk.; M: > 14 drinks/wk)	ϰ	HR (95% CI) Cohort All twins Abstainer .85 (.61,1.20) .79 (.49,1.27) Moderate 1.07 (.79,1.43) .94 (.62,1.42) Heavy 1.08 (.80,1.46) 1.07 (.69,1.66)	**Results suggest a roughly monotonically increasing relationship between alcohol and later receipt of DP due to LBP, with reduced risk for abstainers the clearest feature.**
Ropponen 2014 [52] *see also CVD section	DP due to musculoskeletal diagnoses (MSD) − 5-10 years of follow-up	Twin	Twins with data from a prior study, and at time of that study were living in Sweden, < 65, working and without DP/old age pension; mean age at baseline 53.7 (SD 5.7) ^b^	Swedish Twin Registry	31,206; 922 DP due to MSD-discordant pairs (of which 357 are MZ)	-Co-twin (discordant for DP due to MSD) Cox models and pooled cohort Cox models used to examine risk (stratified for sex in pooled model) -Categories for pooled cohort + twin analyses: abstainers, **light** (≤3 drinks/wk), moderate (F: > 3- ≤ 7 drinks/wk.; M: > 3- ≤ 14 drinks/wk), heavy (F: >7drinks/wk.; M: > 14 drinks/wk)	✓	HR (95% CI) Cohort MZ twins DZ twins All twins Abstainers .93 (.80,1.07) 1.49 (.98,2.26) .8 (.54,1.19) 1.07 (.81,1.42) Moderate **.8** (.69,.93) **2.33 (**1.39,3.91) **.67** (.48,.95) 1.02 (.78,1.34) Heavy **.73** (.67,.81) 1.11 (.82,1.51) **.77** (.60,.98) .88 (.73,1.07)	**Results do not support a clear relationship between alcohol and later receipt of DP due to MSD, particularly given the discrepancy in direction of effects between MZ and DZ twins.**
Ropponen 2011 [58]	DP due to musculoskeletal disorder (MSD) and osteoarthritis specifically (OA) −29 years of follow-up	Twin	MZ and same-sex DZ twins aged ≥18 and working at baseline; mean age 33.2 (SD 12) at baseline	Finnish Twin Cohort	24,043; 1317 pairs discordant for DP due to MSD, 461 pairs discordant for DP due to OA	-Co-twin (discordant for DP due to MSD/OA) Cox models and pooled cohort Cox models used to examine risk for MSD and OA in men and women separately -Categories for pooled cohort + twin analyses: **abstainers**, light (≤3 drinks/wk), moderate (F: > 3- ≤ 7 drinks/wk.; M: > 3- ≤ 14 drinks/wk), heavy (F: >7drinks/wk.; M: > 14 drinks/wk)	ϰ	-For DP due to MSD: HR (95% CI) Cohort (M) Cohort (F) All twins (M) All twins (F) Light .91 (.59,1.40) 1.15 (.93,1.42) **2.04** (1.09,3.82) 1.07 (.81,1.41) Moderate .95 (.70,1.30) 1.23 (1.00,1.51) 1.50 (.94,2.38) .97 (.74,1.27) Heavy 1.01 (.73,1.39) 1.08 (.84,1.39) 1.58 (.99,2.53) 1.37 (.98,1.91) -For DP due to OA specifically: HR (95% CI) Cohort (M) Cohort (F) All twins (M) All twins (F) Light .99 (.50,1.96) 1.19 (.86,1.63) **4.07** (1.15,14.36) 1.33 (.82,2.14) Moderate .78 (.48,1.28) .96 (.69, 1.32) 2.01 (.82,4.93) 1.12 (.70,1.82) Heavy 1.04 (.64,1.71) .86 (.60,1.25) 2.39 (.97,5.89) **2.32** (1.21,4.47)	**Results do not offer enough support for a clear functional form, but are consistent with abstinence representing the lowest risk for DP due to MSD and OA specifically. Heavy drinkers were also at increased risk for both outcomes and sexes.**

^a^ Ropponen et al. (2014) also used this cohort to examine the relationship between alcohol and disability pension due to mental health diagnoses – not reported on here as Samuelsson et al. used the more informative exposure categorization

^b^ From correspondence with authors

Note: Where multiple models are reported in the original paper, the most adjusted analyses are reflected in the above table if (excluding for MR studies)

### Cancer

One twin study reported on two prostate cancer outcomes, prostate cancer and prostate cancer mortality, and results were consistent with reverse J- and J-shaped relationships, respectively, with light drinking at the nadir [43]. Hazard ratios (HRs) for abstainers ranged from 2.85–2.98 (monozygotic (MZ) and combined twin analyses) compared to light drinkers, and for heavy drinkers compared to light drinkers ranged from 1.63–2.00. With the maximum sample (all twin analyses), the confidence interval (CI) for the abstainer comparison was large. Results were similar when restricting analyses to twins discordant for prostate cancer outcome.

### Diabetes

Two studies reported on diabetes, including one twin study, and one MR study. Carlsson et al.’s twin-based findings on the risk for type 2 diabetes (T2D) resemble a J-shape [44], but were critically underpowered, preventing interpretation. In Peng et al.’s MR study, local average treatment effects (LATEs) were employed to detect non-linearity – indicated by a LATE slope (effect of genetically-predicted alcohol consumption plotted against discretized observed alcohol consumption) significantly different to zero [45]. Results did not support non-linearity for diabetes-related biomarkers. Substantive effects were only interpreted for men, with women used as a negative control due to their lack of alcohol consumption (effect of genetic instrument on diabetic markers should be observed in men but not women if alcohol consumption is the only causal pathway). In linear IV analyses, small positive linear relationships with narrow CIs were found for fasting blood glucose (FBG), 2-h post-load plasma glucose (P2hBG) and insulin resistance (HOMA-IR), while there were no relationships for haemoglobin A1c (HbA1c) or beta-cell function (HOMA-beta).

### Dementia

One twin study reported on dementia [46]. Analyses were consistent with a J-shape, with HRs for abstainers compared to light drinkers between 1.37–1.39, and for moderate-to-very-heavy drinkers compared to light drinkers between 1.57–3.07. Analyses of dementia-concordant twins demonstrated that only very heavy alcohol consumers had a much earlier age of onset than their light-drinking co-twins (10.67-year discrepancy in diagnosis compared to 6.79 years when both twins were light drinkers).

### Mental health

Two articles reported on mental health outcomes: a prospective cohort study of depression employing MSMs, and a twin study assessing disability pension due to mental health diagnoses (MHD). Gemes et al. employed DAG-informed MSMs incorporating inverse probability of exposure and attrition weights [47]. Results support a U-shaped relationship (although there were few excessive consumers), with relative risks (RR) of 1.60 and 1.77 for abstainers and excessive drinkers respectively compared to light drinkers. Excluding those with baseline depression increased risk for excessive consumers such that the form approximated a J-shape, with RRs of 1.46 and 2.83 for abstainers and excessive drinkers respectively. Stratifying by gender, only abstainers were at increased risk for men, while both abstainers and excessive consumers remained at increased risk for women.

Samuelsson et al. discretized alcohol consumption into categories based on both volume and frequency [48]. In analyses of outcome-discordant twins, abstainers were at increased risk for pension due to MHD compared to light frequent consumers (HRs of 1.93–2.17 for MZ and combined twin analyses), as were heavy infrequent consumers with an HR of 2.10 (disappeared in MZ-only analyses; HR of 1.09), and light infrequent consumers (HRs of 2.80 and 3.67 for MZ and combined twin analyses respectively). Heavy frequent consumers were at *decreased* risk, although there were too few pairs to conduct MZ-specific analyses.

### Cardiovascular events/diagnoses

Four studies reported on cardiovascular events/diagnoses, including two twin studies, one MSM and one MR study. Ilomaki et al. reported on myocardial infarction (MI), comparing various crude, adjusted and MSM models [49]. The DAG-informed MSM incorporated time-varying consumption and both time-varying and invariant covariates. Results were consistent with a J-shape, with RRs of 1.27 for the lowest group and 1.59 for the highest group respectively, both with fairly narrow CIs (although they included the null). Models 2, 3 and 4 (non-MSM but with assorted incorporation of time-varying exposures/confounders) were consistent with monotonically increasing, reverse-J/U, and J-shaped relationships respectively.

Kadlecova et al. examined the relationship between midlife alcohol consumption and later stroke/transient ischaemic attack (TIA) using MZ twins [50]. In co-twin analyses, all groups had higher odds ratios (ORs) for stroke/TIA than very light consumers, with the highest estimate for abstainers (OR of 2.22; consistent with a reverse J-shape). In twins concordant for stroke/TIA, heavy consumers had shorter time to event (5.68 years), while all other groups had slightly *longer* time to event than the very light drinking group.

Milwood et al. also reported on stroke (ischaemic, intracerebral haemorrhage, total stroke), in addition to acute myocardial infarction (AMI), and total coronary heart disease (CHD) [51]. As for Peng et al., women were negative controls. Comparing categories of genetically-predicted alcohol consumption, MR results were consistent with monotonically increasing relationships for stroke and subtypes, and with no causal relationships for AMI/CHD. Log RRs for those relationships with evidence of linearity ranged from 1.27–1.58 per 280 g of alcohol per week consumed.

Finally, Ropponen et al. reported on disability pension due to circulatory system diagnoses using a twin design [52]. For same-sex twins discordant for outcome, there appeared to be little clear relationship in MZ-only analyses, while the dizygotic (DZ)-only analyses were consistent with a reverse J-shape (both moderate and heavy consumers had HRs < 1 compared to abstainers).

### Continuous cardiovascular measures

Three MR studies reported on lipids, with two of these also reporting on blood pressure and obesity anthropometrics. Peng et al., using the same methods as for diabetes outcomes, found no evidence of non-linear relationships for any lipids, blood pressure measures or obesity anthropometrics, but did find positive linear relationships for BMI, waist circumference, hip circumference, non-HDL-C, triglycerides (TG), total cholesterol (TC), systolic blood pressure (SBP) and diastolic blood pressure (DBP).

Silverwood et al. applied the LATE method to pooled data from 22 studies to examine cardiovascular and inflammatory measures, finding non-linearity (J-shapes) for SBP, non-HDL-C, BMI, WC and C-reactive protein (CRP) [53]. Nadirs for these relationships corresponded to small volumes of alcohol, ranging from 1 to 3.5 units of alcohol/week, and the differences in biomarker outcomes at the nadir compared with abstinence were also small. For those outcomes with no evidence of non-linearity, standard IV analysis revealed a positive linear relationship between alcohol consumption and IL-6 (an inflammatory marker), but a lack of relationship with HDL-C and triglycerides.

Finally, Vu et al. discretized genetically-predicted alcohol consumption into quartiles, comparing lipids in each with the lowest quartile [54]. Results provide evidence of non-linearity for TG, TC, HDL2-C, LDL-C, sdLDL-C and apoB. For these outcomes, all quartiles had more favorable levels than quartile 1. Benefits peaked at quartile 3, equivalent to .5–.1 genetically-predicted units per week. Results do not support causal relationships between alcohol and HDL-C overall, HDL3-C or Lp(a).

### Mortality

One twin study assessed all-cause mortality, finding the three heaviest alcohol consuming groups had greater mortality risk compared to their lighter consuming reference, with HRs ranging from 1.60–2.99 [55]. This pattern replicated in the MZ-only sample, but with less precision and with CIs crossing the null. Abstainers were at decreased risk (HR of .43) in the MZ-only sample, but the CI included the null.

### HIV seroconversion

One study employing MSMs reported on HIV seroconversion in men, incorporating both inverse probability of exposure and censoring weights [56]. Results were consistent with a monotonically increasing risk function, with an RR of 1.61 for heavy drinkers compared with abstainers.

### Musculoskeletal health (MSD)

Three twin studies reported on MSD – all using receipt of disability pension due to MSD conditions as the outcome. Pietikainen et al. found a roughly monotonically increasing relationship for pension due to lower back disorders, with lower risk for abstainers the clearest effect (HR of .79), although all CIs included the null [57]. When stratified by sex, the protective effect of abstinence was more pronounced in men (HR .45; CI .13,1.48), while the functional form in women changed such that there was also reduced risk for moderate consumers (HR .76; CI .45,2.32).

Using the same cohort, Ropponen et al. (2011) examined disability pension due to osteoarthritis and due to MSD more generally [58]. Results were not consistent with a clear functional form but do support abstainers having the lowest risk for both outcomes.

Finally, in a different cohort, Ropponen et al. (2014) evaluated risk for disability pension due to MSD [52]. Again, outcome-discordant twin analyses did not reveal a clear functional form, with discrepant results between MZ and DZ samples.

### Risk of bias

Cohort studies ranged in scores from 7 to 9 out of 9 on the NOS tool (see Table S5), with most losing marks for self-reported ascertainment of exposure. MR studies all had a combination of low and moderate risk across the five domains (see Table S6).

## Discussion

This review found that improved causal inference methods have been applied minimally to research on alcohol–long-term health relationships. Non-linearity was apparent for several outcomes: prostate cancer and related mortality (reverse J-shaped and J-shaped respectively), dementia risk (J-shaped; although age of onset better characterized by monotonically increasing relationship), mental health (U/J-shaped for depression; increased risk for abstainers for disability pension due to MHD), and certain lipids (LDL-C; reverse J-shaped, sdLDL-C and apoB; monotonically decreasing, and HDL-2C; inverted reverse J-shaped). However, many of the individual comparisons from which these overall forms were of small effect size or were imprecise. While the level of consumption coinciding with lowest risk varied between outcomes, it tended to fall in the light range – as little as .5–.1 units/week [54]. Positive linear/monotonically increasing relationships were found for DBP, hip circumference, IL-6, all-cause mortality, and HIV seroconversion (although it is not possible to partition short-term pathways via risky sexual behavior and longer-term effects on immune function). No relationships were found between alcohol and HDL-3C, Lp(a), or waist-to-hip ratio.

Where multiple studies reported on an outcome, findings were inconsistent. For diabetes-related biomarkers, there was a positive linear relationship, but the one study reporting on T2D itself lacked power to support a clear functional form. For cardiovascular events/diagnoses, one twin study found preliminary evidence for a J-shaped relationship with myocardial infarction, while MR failed to find any relationship. For stroke, one twin study found a reverse J-shape (monotonically increasing for time to stroke), while MR found monotonically increasing relationships. For cardiovascular disease more generally, no clear causal relationship emerged. The results of Millwood et al. imply that broad outcomes (in this case total CHD) mask various discrepant sub-functional forms (as is likely for all-cause mortality) [59]. For cardiovascular biomarkers, all three studies evaluating HDL-C were consistent with a lack of causal relationship. There was little consistency across other cardiovascular biomarkers, lipids and obesity anthropometric measures, with conflicting functional forms found for non-HDL-C, triglycerides, total cholesterol, SBP, BMI and waist circumference. This was the case even when the same MR method was used, which may reflect the impact of using different ethnic populations and genetic instruments. Finally, for musculoskeletal health, results varied between a monotonically increasing form, no clear functional form, and no clear functional form with nadir at abstinence.

Some of these findings are roughly consistent with the conclusions on functional form made by recent reviews of the broader observational literature, but there were also outcomes where the present findings do *not* concord with the broader literature; evidence has been triangulated in Table 3. Triangulation with the broader observational research is key, as evidence for most health outcomes was only available from one or two studies in the present review, and thus not definitive.

**Table 3:** Triangulation of included studies with reviews of the broader observational literature

Outcome	Study/ies first author (year)	Simplified summary of findings	Consistent with broader literature?	Details
**Prostate cancer**	Dickerman (2018)	-Reverse J-shaped relationship	No	A 2016 meta-analysis found a monotonically increasing relationship [60]
**All-cause mortality**	Sipila (2016)	-Monotonically increasing	Yes	A 2016 meta-analysis found a monotonically increasing relationship [16]
**Diabetes biomarkers:**				
**–FBG**	Peng (2016)	-Positive linear relationship	Yes	A 2017 review found results consistent with a positive linear relationship [61]
**–Glucose tolerance, insulin sensitivity and glycated haemoglobin**	Peng (2016)	-Positive linear relationships for P2hBG (glucose tolerance) and HOMA-IR (insulin sensitivity) -Lack of relationship for HbA1c (glycated haemoglobin) and HOMA-beta (insulin sensitivity)	N/A	A 2017 review found that, for the other diabetes biomarkers included in this review, there was not enough/consistent evidence from which to draw conclusions [61]
**CVD biomarkers:**				
**–Blood pressure**	Silverwood (2014) Peng (2019)	-Non-linear relationship (small protective effect of light drinking) for SBP -Positive linear relationships for both SBP and DBP	No Yes	A 2018 meta-analysis found no protective effect for hypeternsion [62]
**–Obesity anthropometrics**	Silverwood (2014) Peng (2019)	-Non-linear relationship (small protective effect of light drinking) for BMI and waist circumference -Positive linear relationships for BMI, waist circumference, and hip circumference, with no relationship for waist-to-hip ratio	Mixed Mixed	A 2011 review found mixed evidence on the relationship between alcohol and weight/measures of abdominal adiposity, with heavy drinking generally associated with increased values, but moderate consumption either associated with lower values or not associated at all (suggested that discrepancies may depend on type of alcohol consumed) [63]
**–Inflammatory markers**	Silverwood (2014)	-Non-linear relationship (small protective effect of light drinking) for CRP but a positive linear relationship for IL-6	N/A	A 2021 review of studies from the previous decade found no relevant longitudinal observational studies that were capable of detecting non-linear relationships [64]
**–Lipids: HDL-C**	Silverwood (2014) Peng (2019) Vu (2016)	-No relationship for HDL-C -No relationship for HDL-C -No relationship for HDL-C, or HDL-3C, but an inverted reverse J-shaped relationship for HDL-2C (a beneficial effect)	No No Mixed	A 2021 review of studies from the previous decade found just one longitudinal observational study, which reported a reverse J-shaped relationship in decreases in HDL-C from baseline [64]
**–Lipids: other**	Silverwood (2014) Peng (2019) Vu (2016	-No relationship for TG, but a non-linear relationship (small protective effect of light drinking) for non-HDL-C -Positive linear relationships for TG, non-HDL-C, and TC -Reverse J-shaped relationships for LDL-C, TG and TC, no relationship for Lp(a), and monotonically decreasing relationships for sdLDL-C and apoB	N/A	A 2021 review of studies from the previous decade found no relevant longitudinal observational studies [64]
**CVD events/diagnoses:**				
**–Myocardial infarction**	Ilomaki (2011) Millwood (2019)	-J-shaped relationship -No relationship	No	A 2017 review found alcohol was detrimental for myocarditis [2], but no reviews on myocardial infarction were found
**–Stroke**	Kadlecova (2015) Millwood (2019)	-Reverse J-shaped relationship for stroke and TIA -Monotonically increasing relationships for ischaemic stroke, intracerebral haemorrhage and total stroke	Mixed	A 2016 meta-analysis found J-shaped relationships for ischaemic stroke, but monotonically increasing relationships for intracerebral haemorrhage and subarachnoid haemorrhage [65]
**–Total coronary heart disease**	Millwood (2019)	-No relationship	No	A 2016 meta-analysis found a U-shaped relationship [66]
**–Circulatory system diagnoses**	Ropponen (2014)	-No clear functional form	N/A	Most reviews evaluate CVD sub-conditions, finding divergent functional forms
**Dementia**	Handing (2015)	-J-shaped relationship	Yes	A 2021 review found most recent evidence is consistent with a J-shaped relationship [67]
**Mental health**	Gemes (2019) Samuelsson (2013)	-U−/J-shaped relationship -Non-linear relationship with abstainers at increased risk over light frequent drinkers	Yes	A 2020 meta-analysis found a J-shaped relationship with depressive symptoms [68]
**HIV seroconversion**	Sander (2013)	-Monotonically increasing relationship	N/A	There is a lack of dose-response information on alcohol’s relationship with HIV [69, 70]
**Musculoskeletal disorders**	Pietikainen (2011) Ropponen (2014) Ropponen (2011)	-Monotonically increasing relationship -No clear functional form -No clear functional form, but abstainers have lowest risk	N/A	No reviews on the relationship between alcohol and musculoskeletal disorders were found

Note: Risk for type 2 diabetes (Carlsson et al.) is excluded from this table as the analyses were critically underpowered, and thus the results are not appropriate for inclusion in the triangulation process. Findings from RCTs were not integrated into triangulation as they are almost exclusively conducted in short-term time frames, which is not of interest to this review

Importantly though, where included studies performed conventional analyses for comparison with modern methods to address confounding, results were often discrepant. Most starkly, Millwood et al. found typical J/U/reverse-J-shaped relationships via conventional analyses, but monotonically increasing (or no) relationships when using MR. Even when functional forms roughly replicated across methods, the strength of individual comparisons differed. With pooled cohort analysis, Dickerman et al. found abstainers had slightly increased risk for prostate cancer, compared to much greater risk when utilising discordant twins. The application of improved causal inference methods is therefore essential to accurately characterize alcohol–health relationships.

Also of note, there were several methods of interest for which no eligible studies were identified. It may be that certain methods are ill-suited to address this research question – for example, it may be difficult to find a non-genetic IV to proxy for multiple levels of alcohol consumption, while others, such as G-estimation, may not yet have gained traction in research more generally [21]. Negative controls, while not identified as a primary design approach, were incorporated into two of the MR studies. Of the methods that were represented, there were fewer eligible studies than expected – particularly for MR, where many articles were excluded for only performing linear IV analyses, or for providing estimates in terms of the effects of genetic variants (e.g., ADH1B A-allele carriers vs non-carriers) rather than genetically-predicted alcohol consumption. Consistent with other reviews of the literature [10], covariates controlled for across studies varied considerably (see Table S7).

### Strengths

This review applied a novel framework to examining alcohol–health relationships, identifying and synthesizing information from those observational studies that best mitigate confounding and thus promote causal inference. The search strategy included terms for a broad range of analytical and design-based approaches informed by the literature and consultation with experts. As many included studies performed both conventional analyses and causal inference approaches, this review was able to highlight the difference that such methods make. A further strength was that all long-term health outcomes were eligible, providing a comprehensive picture of the state of the evidence base, and importantly, on the large gaps in the literature where the aforementioned methods require application.

### Limitations

While the included studies mitigate confounding, other methodological limitations (not necessarily captured by the NOS) may be present. For example, the prospective cohort studies likely suffered from sick quitter bias in failing to separate ex-drinkers from lifelong abstainers – exacerbated in those studies where baseline mean age was over 50. Misclassification was likely in many of the twin studies as most based classification on a single measurement, and most of these focused on shared confounding, without additionally controlling for measured covariates. While MSMs can account for consumption and covariates at multiple timepoints, these studies were still vulnerable to residual confounding, with Gemes et al. noting that unmeasured social confounders may partially underpin their findings. As approaches to minimize these biases consist largely of literature-informed, considered researcher decisions and sensitivity analyses, they are not suited to systematic database searching, and were not the focus of this review. While MR is largely immune to both misclassification and confounding, it suffers from its own idiosyncratic limitations, with controversy over its application to alcohol–health research specifically [71–73]. For example, two of the included MR studies discretized genetically-predicted alcohol consumption, resulting in the lowest categories aligning with occasional consumption – not strictly comparable with abstinence.

Additionally, despite evidence of the importance of accounting for pattern of consumption [74], MR studies are limited in their ability to do so [73], and only one cohort study [48] used drinking pattern as the exposure, rather than volume alone (or volume and frequency separately). Finally, several of the included studies evaluated alcohol’s relationship with condition-specific disability pension, rather than the condition itself. This is an imperfect proxy, with receipt of pension also reflecting the interference of the disease with one’s ability to work, as well as incentive to apply [57]. Given that all included studies evaluating musculoskeletal health used disability pension as a proxy, findings with respect to these outcomes should be interpreted with caution.

### Future directions

This review has identified clear gaps in alcohol–long-term health research, demonstrating great potential for further application of enhanced causal inference methods. Analysis methods such as MSMs are particularly promising as they do not require the establishment of twin registries or large genetic datasets, but are able to mitigate confounding, differential censoring and misclassification. And as evidenced by studies included in this review, they are suitable for examining alcohol–long-term health relationships.

Analysis and design approaches that mitigate confounding should be combined with sensitivity analyses such as multiverse analyses (to quantify robustness to data processing/analysis decisions) [75], as well as bias analyses such as e-value generation (to quantify robustness to unmeasured confounding) [76]. Given the unique advantages and limitations of each analytical and design-based approach, triangulation of findings across observational evidence is crucial. Combining data across studies through data harmonization techniques should also be considered to mitigate power issues, which were evident in several included studies with rare exposure–outcome combinations. Finally, while acknowledging the limitations of the included studies, the identification of some evidence consistent with causal protective effects of light-to-moderate alcohol consumption for several health outcomes justifies further exploration of the biological mechanisms that could underpin these (potential) effects. This is particularly true of those outcomes for which findings were concordant with the broader observational literature (see Table 3).

## Conclusions

This novel review found that, when enhanced causal inference approaches are applied, a variety of functional forms – including linear, J-shaped, and no relationship – are found between alcohol consumption and various long-term health outcomes. However, few studies have employed these methods, with covariate-adjusted, conventional cohort analyses remaining dominant, preventing a conclusive picture of the nature of these relationships from emerging. Given that associations found between moderate alcohol consumption and good health impact safe drinking guidelines and public health policy [77, 78], further research employing methods to mitigate confounding and other biases is urgently required to establish whether such findings are truly causal.

## Supplementary Information

**Additional file 1.**

## Data Availability

The datasets used and/or analysed during the current study are available from the corresponding author on reasonable request.

## Abbreviations

RCT: Randomised controlled trial
DAG: Directed acyclic graph
MSM: Marginal structural model
IV: Instrumental variables
MR: Mendelian randomisation
NOS: Newcastle-Ottawa Scale
PRISMA: Preferred Reporting Items for Systematic Review and Meta-Analysis
T2D: Type 2 diabetes
LATE: Local average treatment effects
FBG: Fasting blood glucose
P2hBG: 2-h post-load plasma glucose
HOMA-IR: insulin resistance
HbA1c: Haemoglobin A1c
HOMA-beta: beta-cell function
MHD: Mental health diagnoses
MZ: Monozygotic
MI: Myocardial infarction
TIA: Transient ischaemic attack
AMI: Acute myocardial infarction
CHD: Coronary heart disease
DZ: Dizygotic
TG: Triglycerides
TC: Total cholesterol
SBP: Systolic blood pressure
DBP: Diastolic blood pressure
CRP: C-reactive protein
