# Supplementary material for: Are there non-linear relationships between alcohol consumption and long-term health?: a systematic review of observational studies employing approaches to improve causal inference
Source: BMC Med Res Methodol. 2022 Jan 14;22:16. doi: 10.1186/s12874-021-01486-5 (PMC8759175; doi:10.1186/s12874-021-01486-5)
Supplement: Supplementary file 1 — Additional file 1. [file 12874_2021_1486_MOESM1_ESM.docx]

*Table S1. Ovid MEDLINE Search Strategy*

| 1 exp Alcoholic Beverages/ |
| --- |
| 2 (alcohol* or spirit* or beer* or wine*).mp. |
| 3 exp Alcohol Drinking/ |
| 4 1 or 2 or 3 |
| 5 alcoholic intoxication/ or binge drinking/ |
| 6 Alcohol Abstinence/ |
| 7 Alcohol Dehydrogenase/ |
| 8 aldehyde oxidoreductases/ or aldehyde dehydrogenase/ or aldehyde dehydrogenase 1/ or aldehyde dehydrogenase, mitochondrial/ |
| 9 (alcohol dehydrogenase* or acetaldehyde dehydrogenase* or aldehyde dehydrogenase or ADH1B* or ALDH2* or ADH1C*).mp. |
| 10 ((former or current) adj drinker*).mp. |
| 11 (abstain* or abstinence or "ex drinker*" or "non drinker*" or "non drinking" or exdrinker* or nondrinker* or nondrinking*).mp. |
| 12 ((level* or life-course or lifetime or moderate or regular or low or lower or lowest or light or lighter or lightest or occasional or hazardous or risky or heavy or heavier or heaviest or bing* or episodic or pattern*) adj3 (drinking or drinker* or consum* or intake or "use" or "usage" or habit*)).mp. |
| 13 5 or 6 or 7 or 8 or 9 or 10 or 11 or 12 |
| 14 exp case-control studies/ |
| 15 exp Cohort Studies/ |
| 16 (cohort or case control or longitudinal or prospective or retrospective or follow-up or baseline).mp. |
| 17 14 or 15 or 16 |
| 18 (causal adj2 (effect* or inference or model* or mediat*)).mp. (9656) |
| 19 (mediat* adj2 (pathway* or mechanism*)).mp. |
| 20 Propensity Score/ |
| 21 propensity scor*.mp. |
| 22 (inverse probability or probability weight* or IPW or IPTW).mp. |
| 23 (marginal structural model* or marginal method* or pseudopopulation or pseudo population).mp. |
| 24 (generali#ed method* or G formula* or G estimat* or G computation or G method* or general formula* or structural nested model* or (standardi* and caus*)).mp. |
| 25 (targeted maximum likelihood estimat* or TMLE).mp. |
| 26 Fixed effect* regression.mp. |
| 27 (potential outcome* or doubly robust).mp. |
| 28 or/18-27 |
| 29 negative control.mp. |
| 30 Twin study/ |
| 31 (family-based design or co-relative design or sibship or sibling or twin or genetic epidemiology or genetic variant* or genetically informed method*).mp. |
| 32 Mendelian Randomization Analysis/ |
| 33 (instrumental variable* or instrumental analys* or mendelian randomi*).mp. |
| 34 (Natural experiment or quasiexperiment* or quasi experiment or pre post or "before and after study" or "before and after studies" or before after stud* or difference in differences or interrupted time series or regression discontinuity).mp. |
| 35 or/29-34 |
| 36 35 or (17 and 28) |
| 37 4 and 13 and 36 |
| 38 limit 37 to english language |

*Table S2. Studies contacted for additional information (n=10)*

| **First author (year)** | **Number of contact attempts, dates, author contacted** | **Contact with authors made?** | **Information requested** | **Information received** |
| --- | --- | --- | --- | --- |
|  |  |  |  |  |
| **INCLUDED ARTICLES** | |  |  |  |
| Carlsson (2003) (1) | 1. 22.12.20; lead author | Y; response received 27.12.20 | To confirm that, and ask for reason why, individuals consuming 5-15g/day of alcohol were excluded from discordant twin analyses | Confirmed; in order to ensure twins were truly exposure discordant |
| Peng (2019) (2) | 1. 26.12.20; corresponding author  2. 18.01.21; corresponding author  3. 02.02.21; joint lead author | N | To ask whether Table 4 reports on results adjusted for age only, and to confirm that the LATE method was not applied to diabetes risk itself | N/A |
| Samuelsson 2013 (3) | 1. 04.01.21; corresponding author | Y; response received 05.01.21 | To confirm that reported mean age represents baseline age, and to ask for results from analyses reported as conducted excluding those with baseline mental illness | Confirmed; these analyses were only conducted for the pooled cohort (not discordant twins) and data is not accessible due to COVID |
| Kadlecova (2015) (4) | 1. 11.01.21; corresponding author (bounce back)  2. 11.01.21; other author with accessible contact details – message forwarded to corresponding author | Y; response received 13.01.21 | To confirm that reported twin results are adjusted analyses | Confirmed |
| Silverwood (2014) (5) | 1. 02.02.21; lead author | Y; response received 03.02.21 | To request mean age at baseline for the overall sample | Pointed to another paper (Holmes et al.) containing the same cohorts, from which this statistic can be calculated |
| Ropponen 2014 (6) | 1. 06.02.21; lead author | Y; response received 08.02.21 | To request mean age at baseline for the sample, and to ask whether twin analyses were adjusted for covariates | Mean age was 53.7 (SD 5.7); twin analyses adjusted for sex and age only |
| Sipila 2016 (7) | 1. 20.11.20; corresponding author | Y; response received 20.11.21 | To request information on numbers of drinking discordant pairs | Excel file provided with number of discordant pairs per discordance combination |
| Gemes 2019 (8) | 1. 20.02.21; lead author | Y; response received 20.02.21 | To ask which of two discrepant estimates reported for non-drinker RR is correct | Told that the statistics reported in Table 2 (as opposed to body of text) were correct |
| **ARTICLES DEEMED INELIGIBLE ON BASIS OF AUTHOR RESPONSE** | | |  |  |
| Korhonen 2015 (9) | 1. 06.10.20; lead author  2. 27.10.20; lead author | N | To request more information on twin analyses | N/A |
| Virta 2010 (10) | 1. 06.10.20; lead author  2. 27.10.20; lead author | Y; response received 30.10.21 | To request more information on results of twin analyses | Data no longer accessible |

1. Carlsson S, Hammar N, Grill V, et al. Alcohol Consumption and the Incidence of Type 2 Diabetes A 20-year follow-up of the Finnish Twin Cohort Study. *Diabetes Care*. 2003;26(10):2–7.

2. Peng M, Zhang J, Zeng T, et al. Alcohol consumption and diabetes risk in a Chinese population: a Mendelian randomization analysis. *Addiction*. 2019;114(3):436–449.

3. Samuelsson Å, Ropponen A, Alexanderson K, et al. A prospective cohort study of disability pension due to mental diagnoses : the importance of health factors and behaviors. *BMC Public Health* [electronic article]. 2013;13(1):1. (BMC Public Health)

4. Kadlecová P, Andel R, Mikulík R, et al. Alcohol Consumption at Midlife and Risk of Stroke During 43 Years of Follow-Up: Cohort and Twin Analyses. *Stroke*. 2015;46(3):627–633.

5. Silverwood RJ, Holmes M V, Dale CE, et al. Testing for non-linear causal effects using a binary genotype in a Mendelian randomization study : application to alcohol and cardiovascular traits. *Int. J. Epidemiol.* 2014;(September):1781–1790.

6. Ropponen A, Svedberg P. Single and additive effects of health behaviours on the risk for disability pensions among Swedish twins. *Eur. J. Public Health*. 2013;24(4):643–648.

7. Sipilä P, Rose RJ, Kaprio J. Drinking and mortality : long-term follow-up of drinking- discordant twin pairs. *Addiction*. 2016;111(2):245–254.

8. Gémes K, Forsell Y, Janszky I, et al. Moderate alcohol consumption and depression‐a longitudinal population‐based study in Sweden. *Acta Psychiatr. Scand.* 2019;

9. Korhonen T, Smeds E, Silventoinen K, et al. Cigarette smoking and alcohol use as predictors of disability retirement: a population-based cohort study. *Drug Alcohol Depend.* 2015;155:260–266.

10. Virta JJ, Järvenpää T, Heikkilä K, et al. Midlife alcohol consumption and later risk of cognitive impairment: a twin follow-up study. *J. Alzheimer’s Dis.* 2010;22(3):939–948.

*Table S3. PRISMA checklist*

| **Section/topic** | **#** | **Checklist item** | **Reported on page #** |
| --- | --- | --- | --- |
| **TITLE** | | |  |
| Title | 1 | Identify the report as a systematic review, meta-analysis, or both. | 1 |
| **ABSTRACT** | | |  |
| Structured summary | 2 | Provide a structured summary including, as applicable: background; objectives; data sources; study eligibility criteria, participants, and interventions; study appraisal and synthesis methods; results; limitations; conclusions and implications of key findings; systematic review registration number. | 2 |
| **INTRODUCTION** | | |  |
| Rationale | 3 | Describe the rationale for the review in the context of what is already known. | 3-5 |
| Objectives | 4 | Provide an explicit statement of questions being addressed with reference to participants, interventions, comparisons, outcomes, and study design (PICOS). | 5 |
| **METHODS** | | |  |
| Protocol and registration | 5 | Indicate if a review protocol exists, if and where it can be accessed (e.g., Web address), and, if available, provide registration information including registration number. | 5 |
| Eligibility criteria | 6 | Specify study characteristics (e.g., PICOS, length of follow-up) and report characteristics (e.g., years considered, language, publication status) used as criteria for eligibility, giving rationale. | 5-6 |
| Information sources | 7 | Describe all information sources (e.g., databases with dates of coverage, contact with study authors to identify additional studies) in the search and date last searched. | 5 |
| Search | 8 | Present full electronic search strategy for at least one database, including any limits used, such that it could be repeated. | Table S1 |
| Study selection | 9 | State the process for selecting studies (i.e., screening, eligibility, included in systematic review, and, if applicable, included in the meta-analysis). | 6 |
| Data collection process | 10 | Describe method of data extraction from reports (e.g., piloted forms, independently, in duplicate) and any processes for obtaining and confirming data from investigators. | 6 |
| Data items | 11 | List and define all variables for which data were sought (e.g., PICOS, funding sources) and any assumptions and simplifications made. | 6 |
| Risk of bias in individual studies | 12 | Describe methods used for assessing risk of bias of individual studies (including specification of whether this was done at the study or outcome level), and how this information is to be used in any data synthesis. | 6-7 |
| Summary measures | 13 | State the principal summary measures (e.g., risk ratio, difference in means). | N/A |
| Synthesis of results | 14 | Describe the methods of handling data and combining results of studies, if done, including measures of consistency (e.g., I^2^) for each meta-analysis. | 7 |
| **Section/topic** | **#** | **Checklist item** | **Reported on page #** |
| Risk of bias across studies | 15 | Specify any assessment of risk of bias that may affect the cumulative evidence (e.g., publication bias, selective reporting within studies). | N/A |
| Additional analyses | 16 | Describe methods of additional analyses (e.g., sensitivity or subgroup analyses, meta-regression), if done, indicating which were pre-specified. | N/A |
| **RESULTS** | | |  |
| Study selection | 17 | Give numbers of studies screened, assessed for eligibility, and included in the review, with reasons for exclusions at each stage, ideally with a flow diagram. | 7; Fig. 2 |
| Study characteristics | 18 | For each study, present characteristics for which data were extracted (e.g., study size, PICOS, follow-up period) and provide the citations. | Table 2 |
| Risk of bias within studies | 19 | Present data on risk of bias of each study and, if available, any outcome level assessment (see item 12). | 11; Table S6 |
| Results of individual studies | 20 | For all outcomes considered (benefits or harms), present, for each study: (a) simple summary data for each intervention group (b) effect estimates and confidence intervals, ideally with a forest plot. | Table 2 |
| Synthesis of results | 21 | Present results of each meta-analysis done, including confidence intervals and measures of consistency. | N/A |
| Risk of bias across studies | 22 | Present results of any assessment of risk of bias across studies (see Item 15). | N/A |
| Additional analysis | 23 | Give results of additional analyses, if done (e.g., sensitivity or subgroup analyses, meta-regression [see Item 16]). | N/A |
| **DISCUSSION** | | |  |
| Summary of evidence | 24 | Summarize the main findings including the strength of evidence for each main outcome; consider their relevance to key groups (e.g., healthcare providers, users, and policy makers). | 11-12 |
| Limitations | 25 | Discuss limitations at study and outcome level (e.g., risk of bias), and at review-level (e.g., incomplete retrieval of identified research, reporting bias). | 13 |
| Conclusions | 26 | Provide a general interpretation of the results in the context of other evidence, and implications for future research. | 15 |
| **FUNDING** | | |  |
| Funding | 27 | Describe sources of funding for the systematic review and other support (e.g., supply of data); role of funders for the systematic review. | 1 |

*From:*  Moher D, Liberati A, Tetzlaff J, Altman DG, The PRISMA Group (2009). Preferred Reporting Items for Systematic Reviews and Meta-Analyses: The PRISMA Statement. PLoS Med 6(7): e1000097. doi:10.1371/journal.pmed1000097

*Table S4. Exclusion reasons for key ineligible studies*

Mendelian Randomization studies

| **Study** | **Reason for exclusion** |
| --- | --- |
| Beasley, M., Freidin, M. B., Basu, N., Williams, F. M., & Macfarlane, G. J. (2019). What is the effect of alcohol consumption on the risk of chronic widespread pain? A Mendelian randomisation study using UK Biobank. *Pain*, *160*(2), 501-507. | Could not detect non-linearity. |
| Au Yeung, S. L. A., Jiang, C., Cheng, K. K., Cowling, B. J., Liu, B., Zhang, W., ... & Schooling, C. M. (2013). Moderate alcohol use and cardiovascular disease from Mendelian randomization. *PloS one*, *8*(7), e68054. | Sensitivity analyses excluding heavy drinkers not adequate to determine functional form. |
| Au Yeung, S. L., Jiang, C. Q., Cheng, K. K., Liu, B., Zhang, W. S., Lam, T. H., ... & Schooling, C. M. (2012). Evaluation of moderate alcohol use and cognitive function among men using a Mendelian randomization design in the Guangzhou biobank cohort study. *American journal of epidemiology*, *175*(10), 1021-1028. | Sensitivity analyses excluding heavy drinkers not adequate to determine functional form. |
| Au Yeung, S. L., Jiang, C., Long, M., Cheng, K. K., Liu, B., Zhang, W., ... & Schooling, C. M. (2015). Evaluation of moderate alcohol use with QT Interval and heart rate using Mendelian randomization analysis among older Southern Chinese men in the Guangzhou Biobank Cohort Study. *American journal of epidemiology*, *182*(4), 320-327. | Sensitivity analyses excluding heavy drinkers not adequate to determine functional form. |
| Andrews, S. J., Goate, A., & Anstey, K. J. (2019). Association between alcohol consumption and Alzheimer's disease: A Mendelian Randomization Study. *Alzheimer's & Dementia*. | Could not detect non-linearity. |
| Holmes, M. V., Dale, C. E., Zuccolo, L., Silverwood, R. J., Guo, Y., Ye, Z., ... & Langenberg, C. (2014). Association between alcohol and cardiovascular disease: Mendelian randomisation analysis based on individual participant data. *Bmj*, *349*, g4164. | Did not conduct formal IV analysis/provide estimates in terms of genetically-predicted alcohol consumption. |
| Kumari, M., Holmes, M. V., Dale, C. E., Hubacek, J. A., Palmer, T. M., Pikhart, H., ... & Bobak, M. (2014). Alcohol consumption and cognitive performance: a Mendelian randomization study. *Addiction*, *109*(9), 1462-1471. | Sensitivity analyses excluding heavy drinkers not adequate to determine functional form. |
| Lawlor, D. A., Nordestgaard, B. G., Benn, M., Zuccolo, L., Tybjaerg-Hansen, A., & Davey Smith, G. (2013). Exploring causal associations between alcohol and coronary heart disease risk factors: findings from a Mendelian randomization study in the Copenhagen General Population Study. *European heart journal*, *34*(32), 2519-2528. | Could not detect non-linearity. |
| Tabara, Y., Arai, H., Hirao, Y., Takahashi, Y., Setoh, K., Kawaguchi, T., ... & Nagahama Study Group. (2017). The causal effects of alcohol on lipoprotein subfraction and triglyceride levels using a Mendelian randomization analysis: The Nagahama study. *Atherosclerosis*, *257*, 22-28. | Could not detect non-linearity. |

Cohort studies

| **Study** | **Reason for exclusion** |
| --- | --- |
| Koch, M., Costanzo, S., Fitzpatrick, A. L., Lopez, O. L., DeKosky, S., Kuller, L. H., ... & Mukamal, K. J. (2020). Alcohol Consumption, Brain Amyloid-β Deposition, and Brain Structural Integrity Among Older Adults Free of Dementia. *Journal of Alzheimer's Disease*, (Preprint), 1-11. | IPWs used for attrition only. |
| Paul, K. C., Chuang, Y. H., Shih, I. F., Keener, A., Bordelon, Y., Bronstein, J. M., & Ritz, B. (2019). The association between lifestyle factors and Parkinson's disease progression and mortality. *Movement Disorders*, *34*(1), 58-66. | IPWs used for attrition only. |
| Marti, C. N., Choi, N. G., DiNitto, D. M., & Choi, B. Y. (2015). Associations of lifetime abstention and past and current alcohol use with late-life mental health: a propensity score analysis. *Drug and alcohol dependence*, *149*, 245-251. | Cross-sectional. |
| Kerr, W. C., & Ye, Y. (2010). Relationship of life-course drinking patterns to diabetes, heart problems, and hypertension among those 40 and older in the 2005 US National Alcohol Survey. *Journal of studies on alcohol and drugs*, *71*(4), 515-525. | Cross-sectional. |
| Beulens, J. W., van der Schouw, Y. T., Moons, K. G., Boshuizen, H. C., & Groenwold, R. H. (2013). Estimating the mediating effect of different biomarkers on the relation of alcohol consumption with the risk of type 2 diabetes. *Annals of epidemiology*, *23*(4), 193-197. | Exposure was not categorized, and so study could not detect non-linearity. |

*Table S5. Risk of Bias in cohort studies as assessed by the Newcastle-Ottawa Scale*

| **Study first author** | **Selection 1** | **Selection 2** | **Selection 3** | **Selection 4** | **Comparability** | **Outcome 1** | **Outcome 2** | **Outcome 3** | **Total /9** |
| --- | --- | --- | --- | --- | --- | --- | --- | --- | --- |
| Carlsson | **🟑** | **🟑** |  | **🟑** | **🟑🟑** | **🟑** | **🟑** | **🟑** | 8 |
| Dickerman | **🟑** | **🟑** |  |  | **🟑🟑** | **🟑** | **🟑** | **🟑** | 7 |
| Gemes | **🟑** | **🟑** |  | **🟑** | **🟑🟑** |  | **🟑** | **🟑** | 7 |
| Handing | **🟑** | **🟑** |  | **🟑** | **🟑🟑** | ^a^ | **🟑** | **🟑** | 7 |
| Ilomaki | **🟑** | **🟑** |  |  | **🟑🟑** | **🟑** | **🟑** | **🟑** | 7 |
| Kadlecova | **🟑** | **🟑** |  | **🟑** | **🟑🟑** | **🟑** | **🟑** | **🟑** | 8 |
| Pietkainnen | **🟑** | **🟑** |  | **🟑** | **🟑🟑** | **🟑** | **🟑** | **🟑** | 8 |
| Ropponen (2011) | **🟑** | **🟑** |  | **🟑** | **🟑🟑** | **🟑** | **🟑** | **🟑** | 8 |
| Ropponen (2014) | **🟑** | **🟑** | **🟑** | **🟑** | **🟑🟑** | **🟑** | **🟑** | **🟑** | 9 |
| Samuelsson | **🟑** | **🟑** | **🟑** | **🟑** | **🟑🟑** | **🟑** | **🟑** | **🟑** | 9 |
| Sander | **🟑** | **🟑** | ^b^ | **🟑** | **🟑🟑** | **🟑** | **🟑** | **🟑** | 8 |
| Sipila | **🟑** | **🟑** |  | **🟑** | **🟑🟑** | **🟑** | **🟑** | **🟑** | 8 |

Selection 1 = representativeness of the exposed cohort [star for truly or somewhat representative]

Selection 2 = selection of the non-exposed cohort [star for drawn from same community as exposed cohort]

Selection 3 = ascertainment of exposure [star for secure record or structured interview]

Selection 4 = demonstration that outcome of interest was not present at start of study [star for yes]

Comparability = comparability of cohorts on the bases of the design or analysis [up to two stars for controlling for the most important factor and any additional important factor]

Outcome 1 = assessment of outcome [star for independent blind assessment or record linkage]

Outcome 2 = was follow-up long enough for outcomes to occur [star for yes]

Outcome 3 = adequacy of follow-up cohorts [star for complete follow-up or subjects lost to follow-up unlikely to introduce bias]

^a^ Study did use record linkage but had poor sensitivity.

^b^ Study used a mix of self-report and structured interview, but for some participants only self-report was conducted (depending on time of enrolment).

*Table S6. Risk of Bias in Mendelian Randomization studies using Mamluk et al. tool* (1)

| **Study first author** | **Weak instrument bias** | **Genetic confounding** | **‘Non-genetic confounding’^a^** | **Pleiotropy** | **Selection Bias** |
| --- | --- | --- | --- | --- | --- |
| Silverwood | Moderate risk | Moderate risk^b^ | Low risk | Moderate risk | Moderate risk |
| Millwood | Moderate risk | Low risk | Low risk^c^ | Low risk | Low risk^d^ |
| Peng | Low risk | Low risk | Low risk | Low risk | Moderate risk |
| Vu | Moderate risk^e^ | Low risk | Low risk | Low risk | Low risk |

^a^ Inclusion of covariates other than age or sex in analyses.

^b^ Although simulations demonstrated robustness of relationship detection to confounding.

^c^ Further analyses did adjust for education, income and smoking, but we did not extract these given the potential for introducing bias.

^d^ While the study did not adjust for principal components, it did stratify by areas within China.

^e^ Some F values slightly above and some slightly below 10.

1. Mamluk L, Jones T, Ijaz S, et al. Evidence of detrimental effects of prenatal alcohol exposure on offspring birthweight and neurodevelopment from a systematic review of quasi-experimental studies. *Int. J. ofEpidemiology*. 2020;1–24.

*Table S7. Covariates adjusted/stratified for in each study*

| **Study first author** | **Main causal analyses** | **Main conventional analyses (if applicable)** | **Secondary/sensitivity causal (if applicable)** | **Secondary/sensitivity other (if applicable)** |
| --- | --- | --- | --- | --- |
| Dickerman | Twin: none | Intrapair correlations (for SEs/CIs only), BMI, smoking, social class, education, physical activity | N/A | N/A |
| Carlsson | Twin: none | Age, BMI | N/A | Smoking, socioeconomic status, physical activity, intrapair correlations |
| Peng | MR: age, sex | N/A | Age, sex, BMI, waist circumference, physical activity, and education (results of these analyses not extracted in this review as controlling for additional confounders in MR studies can introduce bias) | N/A |
| Handing | Twin: age, sex, education, smoking, physical activity, diabetes, and hypertension | Age, sex, education, smoking, physical activity, diabetes, and hypertension | N/A | N/A |
| Gemes | MSM: alcohol consumption pre-baseline, symptoms of depression, stressful life events, financial stress, social support, and having small children measured both pre-baseline and at baseline considered as time-varying confounders; age, sex, education, country of birth, childhood adversities, smoking included as time-fixed confounders (assessed only at baseline) | Non-MSM: age, sex, education, being born in Sweden, cohabiting with small children, smoking, stressful life events, financial stress, childhood adversities, and baseline MDI | MSM without those with baseline depression: living with small children, stressful life events, financial stress, social support, symptoms of depression and previous alcohol consumption as time-varying covariates; age, sex, education, country of birth, smoking and childhood adversities as time-stable covariates | Non-MSM without those with baseline depression: age, sex, education, being born in Sweden, cohabiting with small children, smoking, stressful life events, financial stress and childhood adversities |
| Samuelsson | Twins: age and sex | Age, sex, education, marital status, disease severity, physical activity, BMI, tobacco use, and self-rated health | N/A | N/A |
| Ilomaki | MSM: time-invariant (age, marital status, rurality, employment status, education, family history of CVD, cigarettes per day, years of smoking, and diabetes) and time-varying covariates (BMI, smoking status, HDL-C, serum insulin, serum fibrinogen, resting SBP, and history of CVD) | Various – see Table 1 in paper proper | N/A | N/A |
| Kadlecova | Twin: age, sex, preferred alcohol, current smoking, BMI, physical activity, stress reactivity, and a range of other illnesses | Age, sex, preferred alcohol, current smoking, BMI, physical activity, stress reactivity, and a range of other illnesses | Age, sex, preferred alcohol, ever smoking, BMI, physical activity, stress reactivity, and a range of other illnesses | Age, sex, preferred alcohol, ever smoking, BMI, physical activity, stress reactivity, and a range of other illnesses |
| Millwood | MR: area and age | Area, age, education, household income, and smoking | Area, age, education, income, and smoking (results of these analyses not extracted in this review as controlling for additional confounders in MR studies can introduce bias) | N/A |
| Ropponen (2014) | Twins: age and sex | Stratified by sex and clustered on twin-pair identify; adjusted for age, BMI, other diseases, education, marital status, and health behaviours | N/A | N/A |
| Silverwood | MR: age, sex, and study | N/A | N/A | N/A |
| Vu | MR: sex, age, and genetic principal components | None reported | N/A | N/A |
| Sipila | Twin: age, gender, marital status, smoking status, physical activity, obesity, education, and social class | Age, gender, marital status, smoking status, physical activity, obesity, education, and social class | N/A | N/A |
| Sander | MSM: race, ethnicity, age, enrollment city, and education as time-fixed covariates; depressive symptoms, gonorrhea/chlamydia, smoking, and illicit drug use as time-dependent covariates lagged one visit | Baseline covariates: age (spline), race, ethnicity, education; time-dependent covariates lagged one visit: illicit drug use, cigarette smoking, depression, sexually transmitted infection, and multiple unprotected receptive anal intercourse partners | N/A | N/A |
| Pietikainen | Twin: age | Age | N/A | N/A |
| Ropponen (2011) | Twin: none | Age, socio-demographic factors, education, social class, health-related factors, BMI, chronic disease, number locations musculoskeletal pain, use of analgesiscs, smoking status, and clustered on twin pair identity | N/A | N/A |
